# A High-Resolution Luminescent Assay for Rapid and Continuous Monitoring of Protein Translocation across Biological Membranes

**DOI:** 10.1016/j.jmb.2019.03.007

**Published:** 2019-04-05

**Authors:** Gonçalo C. Pereira, William J. Allen, Daniel W. Watkins, Lisa Buddrus, Dylan Noone, Xia Liu, Andrew P. Richardson, Agnieszka Chacinska, Ian Collinson

**Affiliations:** 1 School of Biochemistry, University of Bristol, Bristol, UK; 2 BrisSynBio, University of Bristol, Bristol, UK; 3 Centre of New Technologies, University of Warsaw, S. Banacha 2c, 02-097, Warsaw, Poland

**Keywords:** AA, antimycin A, IMM, inner mitochondrial membrane, mt-11S, mitochondrial-targeted 11S, _H6_11S, N-terminal 6xHis tagged version of 11S, OMM, outer mitochondrial membrane, OVA, oligomycin, valinomycin and antimycin A (full depolarization cocktail), MTS, mitochondrial targeting sequence, NanoBiT, NanoLuc Binary Technology, PAM, presequence-translocase-associated import-motor, pep86, high-affinity version of NanoBiT small fragment, pep114, small fragment of NanoBiT, PMF, proton motive force, TIM, translocase of the inner membrane, TOM, translocase of the outer membrane, 11S, large fragment of the NanoBiT, ΔΨ, membrane potential, protein translocation, mitochondrial protein import, bacterial Sec system, NanoLuc, live assay

## Abstract

Protein translocation is a fundamental process in biology. Major gaps in our understanding of this process arise due the poor sensitivity, low time resolution and irreproducibility of translocation assays. To address this, we applied NanoLuc split-luciferase to produce a new strategy for measuring protein transport. The system reduces the timescale of data collection from days to minutes and allows for continuous acquisition with a time resolution in the order of seconds, yielding kinetics parameters suitable for mechanistic elucidation and mathematical fitting. To demonstrate its versatility, we implemented and validated the assay *in vitro* and *in vivo* for the bacterial Sec system and the mitochondrial protein import apparatus. Overall, this technology represents a major step forward, providing a powerful new tool for fundamental mechanistic enquiry of protein translocation and for inhibitor (drug) screening, with an intensity and rigor unattainable through classical methods.

## Highlights

• Conventional methods for monitoring protein translocation are laborious and discontinuous and lack kinetic detail.
• A split NanoLuc system was adapted for real-time monitoring of protein translocation through the bacterial Sec system and the mitochondrial Tim23 complex.
• The new assay reduces the timescale of data acquisition from days to minutes.
• It produces high-quality results suitable for kinetic fitting and model derivation.

## Graphical Abstract

**Figure:**
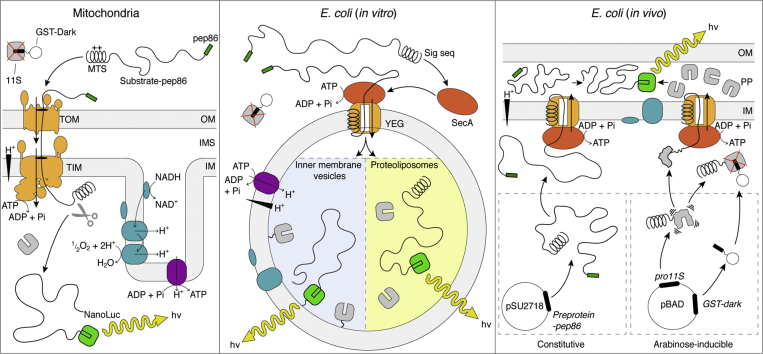

## Introduction

A large proportion of proteins fulfil their function outside the cell, or in subcellular compartments distinct from the cytosol. To get there, they must be sorted and then transported across the appropriate membranes; thus, protein translocation systems are ubiquitous and fundamental features of cellular compartmentalization. For example, bacteria target about 20% of their proteome to the Sec system for transport across or into the plasma membrane [1], while nearly all mitochondrial proteins are produced in the cytosol for import through the Translocases of the Outer and Inner Membranes (TOM and TIM). In both of these cases, proteins are recognized by specific targeting sequences that are often cleaved upon completion of transport to liberate the mature protein.

Previously, protein translocation has been monitored *in vitro* by quantifying time courses of proteins transported into the interior of reconstituted proteoliposomes (PLs) or native membranes, for example, bacterial inner-membrane vesicles [2], [3] (IMVs) or intact mitochondria [4], [5]. In these experiments, successfully transported protein is typically characterized by resistance to proteolysis and detected by Western blotting or autoradiography. These classical methods have been instrumental for the determination of the molecular components and basic properties of the various prokaryotic [6] and eukaryotic translocation apparatus [7], [8]. However, such assays are not suited to a more sophisticated analysis, due to lack of kinetic detail—they produce only discontinuous, end-point measurements—and are labor intense, making them difficult to scale to high throughput. Over the past two decades, various alternative methods have been proposed (Table S1), all of which have drawbacks. A highly sensitive, versatile and quantitative real-time assay has yet to be developed.

Recently, Dixon *et al*. [9] developed a non-covalent complementation system based on a small, bright luciferase, to monitor protein–protein interactions—NanoBiT (short for NanoLuc Binary Technology). In this split-luciferase, the final β-strand of NanoLuc was cleaved to generate a large fragment of 18 kDa, referred to as *11S* (trademark name *LgBiT*), and a small 1.3-kDa peptide chain of 11 amino acids, termed *pep114* (trademark name *SmBiT*). The authors also developed a high-affinity variant of pep114, *pep86* (trademark name *HiBiT*; picomolar range); here, we exploit the rapid, spontaneous interaction between 11S and pep86 as the basis for a protein translocation assay.

Using simple genetic tools, we targeted 11S to two model destination compartments: the yeast mitochondrial matrix and the *Escherichia coli* periplasm (Fig. S1a–c). The presence of lipid bilayers keeps the reporter segregated, ensuring that complementation is restricted to the destination cell compartment. Substrate pre-proteins, that is, proteins with their targeting sequence uncleaved, are then tagged with pep86 at their C-terminus. Upon translocation, the rise in local protein concentration leads to complementation of pep86 with internalized 11S, producing active luciferase activity and thus a readout for protein translocation. Pep86 is small and native-like, so should eliminate artifacts caused by non-native tags such as fluorescent dyes, and has negligible effect on transport rates. The enzymatic amplification generated by NanoLuc, meanwhile, provides very high-sensitivity measurements compared to conventional methods (Table S1), allowing for detailed quantitative analyses of translocation. We anticipate that the techniques and tools described here will be readily transferrable to many other membrane and non-membrane protein translocation systems.

## Results

### *In vitro* continuous translocation assay of the bacterial Sec machinery

**Fig. 1:**
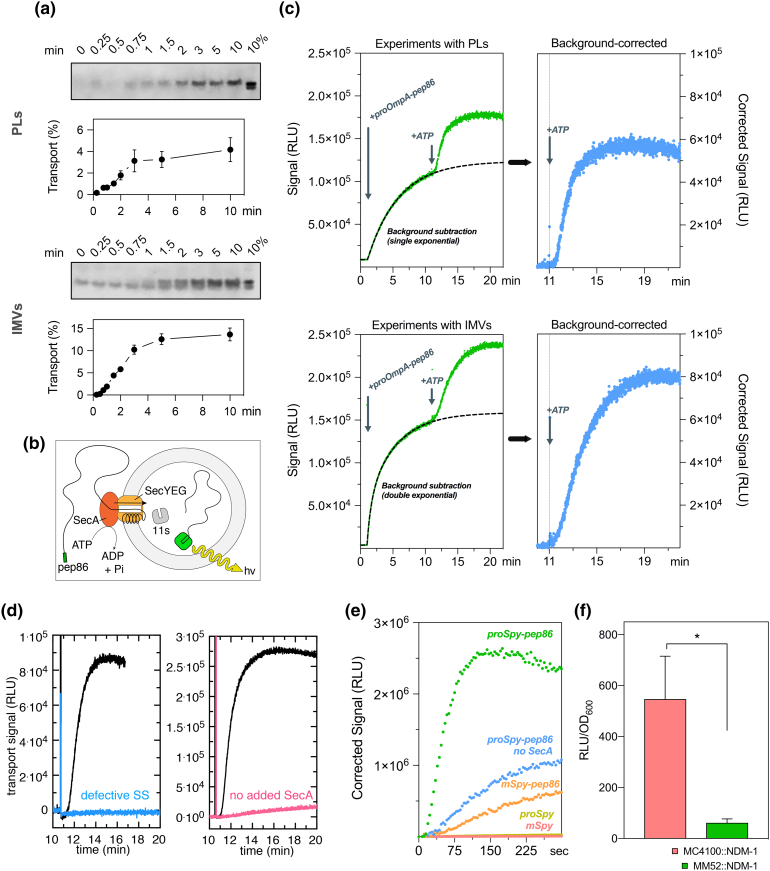
Comparison of conventional *versus* real-time translocation assay for the bacterial SecYEG sytem. (a) *In vitro* reactions in PLs (top) and IMVs (bottom) were carried out in the presence of an ATP regenerating system, SecA and 200 nM of proOmpA; reaction started by addition of 1 mM ATP. At the indicated timepoints, an aliquot was withdrawn and quickly quenched by dilution in ice-cold protease K and EDTA. Protease-protected OmpA was evaluated by SDS-PAGE followed by immunoblotting. (b) Diagram explaining the concept behind the real-time import assay (a more detailed version is available in Fig. S1b). (c) For the real-time translocation assay, 11S (20 μM) was included in the buffers for PL preparation ensuring its encapsulation (top) or tethered to the inner membrane of HB1 cells (IMVs; bottom). Reactions were carried out on a cuvette luminometer with identical buffers as described before. Reactions were started by the addition of proOmpA in the absence of ATP, and the luminescence background was monitored, and then ATP was added to start pre-protein translocation. (d) Real-time import of competent (black trace) or incompetent (defective signal sequence; blue trace) proOmpA-pep86 into PLs is shown on the left-hand side. Real-time import of proOmpA-pep86 with (black trace) and without SecA (coral trace) is shown on the right-hand side. Uncorrected traces can be found in the supplemental material (Fig. S2c, d). (e) Real-time import of proSpy-pep86 into BL21(DE3) IMVs containing 11S. Reactions were carried out as described in panel a but on a 96-well plate format. Plot represents the signal after background is omitted from the graph. Deletion of the signal sequence (mSpy-pep86) or absence of SecA prevented import of pre-protein. No signal is observed if pep86 is absent from the pre-protein. (f) Evaluation of NDM-1-pep86 translocation *in vivo* by co-expressing periplasm-targeted 11S; end-point measurements were taken after a 2-h induction and compared to a SecA temperature-sensitive mutant (MM52 strain) and normalized to cell number. Abbreviations: IMVs, inner-membrane vesicles; PLs, proteoliposomes.

The bacterial Sec system is the principal mechanism for protein secretion across the bacterial plasma membrane, and a good starting point for assay development as its activity has been extensively studied. Transport through the Sec machinery can be recapitulated *in vitro* using only a small number of purified components: PLs [6] or IMVs [10], [11] containing the SecYEG protein–channel membrane protein complex, the soluble motor ATPase SecA, a pre-secretory protein substrate with a cleavable N-terminal signal sequence, and ATP as an energy source. In both cases, successful translocation results in internalization of pre-protein, equivalent to translocation into the periplasm *in vivo*. When the reaction is complete, protease K is added to digest untranslocated pre-protein, while the vesicle protects the successfully translocated material. Alternatively, samples can be taken from the reaction at various time points and quenched with ice-cold buffer containing protease K, to investigate the transport kinetics. Analysis is typically performed by SDS-PAGE followed by autoradiography (of radiolabeled substrates) or immunoblotting (Fig. 1a).

These assays do not enable an exploration beyond a simple analysis, so we set out to modify SecYEG PLs, IMVs and substrate pre-proteins such that they would be compatible with a split NanoLuc-based assay (Figs. 1b and S1b). For PLs, we included purified _6H_11S into the reconstitution mixture and then removed excess protein by three rounds of centrifugation and resuspension in clean buffer, to reduce the background signal (Fig. S2a, b). To ensure a high concentration of internalized 11S within IMVs, we produced a variant of 11S with an inner-membrane lipid anchor sequence [12]. We then co-transformed the expression vectors harboring 11S and SecYEG into *E. coli* and induced expression of both plasmids simultaneously. Pre-proteins were extended at the C-terminus by a GSG linker followed by pep86.

Reactions were initially set up with either PLs or IMVs saturated with SecA together with an ATP regeneration system, Prionex—a biocompatible polymer used for protein stabilisation and to prevent 11S/pre-protein-pep86 from adhering to surfaces—and the NanoLuc substrate furimazine. Luminescence measurements were then started to generate a baseline reading, followed by the addition of the model pre-protein, proOmpA, fused to pep86 (Fig. 1c).

The initial experiments were prone to a high, ATP-independent luminescence signal, presumably due to contaminating external 11S (Fig. S2a), remnants of the reconstitution or from burst vesicles during handling. This problem was resolved by supplementing the mixture with an inactivated form of the pep86, “*dark*” peptide [13]. This peptide differs from the pep86 sequence by a point mutation at a critical catalytic arginine residue, which does not prevent high-affinity binding to 11S but prevents catalysis once bound. For ease of production, we synthesized a fusion of glutathione *S*-transferase and the “*dark*” peptide, which we named *GST-dark*. The inclusion of 40 μM GST-dark did indeed massively reduce, but did not completely obviate, the background signal contribution (Figs. 1c and S2b). The remaining background signal could be fitted to either a single or double exponential function and subtracted from the data acquired after the addition of ATP (Fig. 1c).

The high detail of the background-corrected transport traces is clear (Fig. 1c). To confirm that the data report on the kinetics of translocation rather than slow rate-limiting pep86–11S association, we performed further assays with PLs containing a range of 11S concentrations (Fig. S3a). The results show that while the signal amplitude is proportional to the concentration of 11S—that is, the reaction ends when all 11S associates with pre-protein-pep86—the shape of the curve is completely unaffected (Fig. S3b). Thus, rates extracted from the data do indeed reflect the rate of transport. Omission of SecA from the system abolished the signal, demonstrating that proOmpA-pep86 translocation into PLs was ATP- and SecA-dependent (Figs. 1d and S2c). Similarly, deletion of four hydrophobic amino acids (IAIA) in the signal sequence of proOmpA-pep86, which prevents the export of pre-proteins *in vivo* [14], stops its import into PLs (Figs. 1d and S2d).

To demonstrate the versatility of the assay, we also linked the pep86 sequence to the C-terminus of a very different *E. coli* pre-protein—the soluble, positively charged and α-helical spheroplast protein Y (Spy), for comparison against the β-barreled proOmpA. The translocation data for proSpy (Fig. 1e, green trace) are qualitatively similar to those of proOmpA, demonstrating the broad compatibility of the assay. Importantly, removal of either the signal sequence or pep86 from proSpy, or SecA from the reaction mixture collapsed the signal. The omission of SecA from experiments conducted with IMVs retained residual activity (Fig. 1e, blue trace), probably due to contamination by endogenous membrane-associated SecA [15]. Mature Spy-pep86 also appears to retain a residual, ATP-dependent translocation competence, which could be due to a cryptic signal sequence or mature domain Sec targeting factors [16].

Taken together, the results show that the luminescence signal is a *bona fide* measure of protein transport and suitable for a comprehensive kinetic analysis of the ATP and proton motive force (PMF)-driven secretion process—to be described in forthcoming publications.

### *In vivo* β-lactamase secretion assay

Next, we set out to design a split-NanoLuc-based system for measuring translocation through the bacterial translocon *in vivo* (Fig. S1c)*.* For this, we attached pep86 to the pre-secretory protein *N*ew *D*elhi *m*etallo-beta-lactamase 1 (NDM1), a protein of great current interest due to its involvement in mediating antibiotic resistance in hospitals [17]. We also constructed an 11S variant with the N-terminal signal sequence of proOmpA (forming pro-11S), directing it to the periplasm. To reduce the background from newly synthesized pro-11S that has yet to be secreted, *GST-dark* was expressed in the cytoplasm (Fig. S4). By comparing an *E. coli* strain with severe secretion defects [18] to its parent strain, we show that this system does indeed produce a secretion-dependent luminescence signal (Fig. 1f). This *in vivo* measure of secretion will be a powerful tool for understanding the native secretion process as well as for the development of new screens for antibiotic discovery and development of strategies against *a*nti-*m*icrobial *r*esistance.

### Real-time import assay in isolated yeast mitochondria

**Fig. 2:**
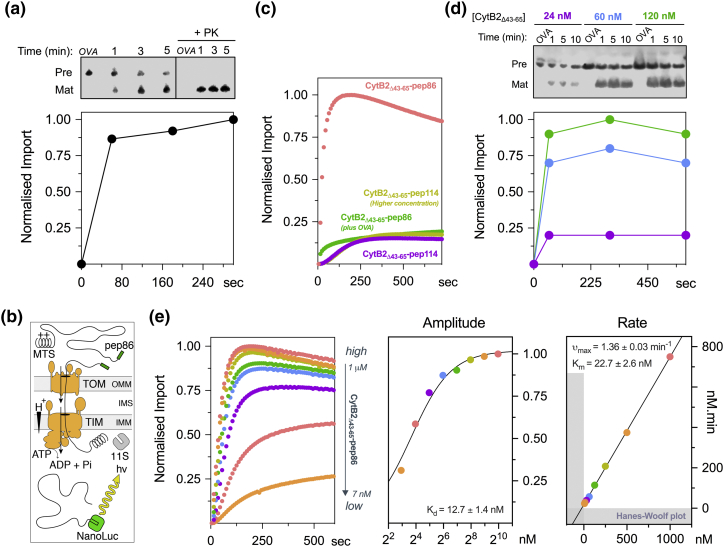
Comparison between the conventional method and the new real-time import assay to monitor protein import in mitochondria. (a) Conventional method: fully energized mitochondria were challenged with 62 nM CytB2_Δ43–65_-pep86 and aliquots collected and quenched at specific timepoints. To identify successful translocation, samples were digested with protease K, and mature (cleaved signal sequence) protein was identified by immunoblotting after SDS-PAGE. (b) Diagram explaining the concept behind the real-time import assay (a more detailed version is available in Fig. S1a). (c) Typical real-time import traces where fully energized mt-11S mitochondria were challenged with 1 μM CytB2_Δ43–65_-pep86 in the presence or absence of OVA. If the low-affinity pep114 tag was used instead, no significant gain-of-signal was observed (1 μM, low; 10 μM, high). Typical time course of dose–response of CytB2_Δ43–65_-pep86 using the conventional (d) *versus* the new real-time assay (e). The right side of panel e represents parameters obtained from the normalized traces. The rate (*k*_app_) was calculated as the inverse of the time it takes to reach half of the maximal signal (*t*_50%_ in min). Abbreviations: OVA, oligomycin, valinomycin and antimycin cocktail.

Over the past 40 years, isolated mitochondrial fractions of *Saccharomyces cerevisiae* have been used widely to study protein translocation *in vitro*. Since tools for genetic engineering of yeast are widespread and large quantities of the organism can be grown to yield considerable amounts of mitochondria, yeast mitochondria are a perfect candidate for applying split-NanoLuc to a eukaryotic protein translocation system (Fig. 2b and Fig. S1a).

Classical mitochondrial import assays employ autoradiography or Western blotting (Fig. 2a) to detect transport. Similar to the bacterial setup, reaction aliquots representing time points are quenched by moving samples to ice followed by addition of a “death” cocktail of oligomycin, valinomycin and antimycin A (OVA) and protease K, to respectively collapse the PMF and digest non-translocated precursors. Import is indicated by detection of the mature protein, that is, after cleavage of the mitochondrial targeting sequence (MTS), by SDS-PAGE followed by immunoblotting. As shown in Fig. 2a, the data points are discrete with a time resolution of the order of minutes.

To develop the new transport assay (Fig. 2b), we targeted 11S to the mitochondrial matrix using the presequence of subunit F_1_α of the yeast ATP synthase (mt-11S). After mitochondrial isolation, samples were analyzed by SDS-PAGE to confirm localization of 11S to the matrix (Fig. S5a). The mass of mt-11S is identical to that of purified _H6_11S, suggesting efficient localization to the matrix, where the MTS is removed. Under standard culture conditions, 1% galactose resulted in 1.34 ± 0.05 μM of mature 11S in mitochondria, assuming a standard matrix volume of 1.1 μL/mg of protein [19] and using pure _H6_11S as a standard (Fig. S5b, c). This could be useful for determining absolute quantities of imported protein (Fig. S5d, f).

As an import substrate, we used the classical yeast import precursor cytochrome B2 (CytB2; YML054C) with its hydrophobic sorting domain removed (CytB2_Δ43–65_), causing it to localize to the matrix [20]. We found that standard *in vitro* import reaction conditions [4], [8], [21] required optimization: but by lowering both the concentration of mitochondria and of furimazine, we were able to produce a strong, transport-dependent luminescence signal, which could be maintained for minutes (Fig. S6).

A typical optimized import assay trace is shown in Fig. 2c. It comprises a baseline corresponding to the background produced by 11S alone (omitted from the graph) followed by a sigmoidal shape upon CytB2_Δ43–65_-pep86 addition. No increase in signal was observed if the low-affinity tag pep114 was used, confirming that spontaneous complementation is necessary to report translocation (Fig. 2c, purple trace). In a separate experiment, mitochondrial respiration was inhibited, and membrane potential (ΔΨ) dissipated by addition of the OVA “death” cocktail (Fig. 2c, green trace). Under these conditions, no rise in luminescence was observed upon the addition of CytB2_Δ43–65_-pep86, confirming that the signal reflects energy-dependent import. Note that background luminescence in the presence of OVA was significantly higher than the baseline (~ 12%)—albeit without the characteristic sigmoidal shape (Fig. S7)—suggesting that import-independent complementation is due to leakage of 11S from broken mitochondria that is not fully “quenched” by GST-dark (Fig. S8a–f).

To eliminate the possibility of mitochondrial poisons non-specifically interfering with the assay, we performed binding assays in the presence of low and high concentration of these drugs (Table S2). This was the case only for CCCP, which at 1 μM lowered the maximum amplitude to 40% if pre-incubated. Therefore, CCCP was not used from here onward.

Next, we utilized the high-throughput capabilities of the assay and monitored CytB2_Δ43–65_-pep86 import over a range of concentrations. Attempts to carry out a similar titration assay using the conventional method have failed to deliver reliable results (Fig. 2d), due to the limited range of concentrations that can be practically measured (~ 24–120 nM). By contrast, the split-luciferase assay produces a dose–response curves of CytB2_Δ43–65_-pep86 spanning 2 orders of magnitude (7–1000 nM), with sufficient data quality to extract kinetic parameters (Fig. 2e). To ensure that the observed kinetics report on import, rather than slow rate-limiting complementation of the reporter, we performed binding experiments with CytB2_Δ43–65_-pep86 and _6H_11S alone. Although linking the pep86 tag to a precursor generally decreased its affinity for 11S, the *Κ*_d_ remains sufficiently low (33.5 ± 5.7 nM; Fig. S9) compared to the effective mt-11S concentration (1.34 ± 0.05 μM). Given that association of CytB2_Δ43–65_-pep86 and 11S is much faster than the rate of translocation (*ν*_max_ in Fig. S9 *versus* Fig. 2e; 13.5 ± 0.7 min^−1^ *versus* 1.36 ± 0.03 min^−1^, respectively), we can be assured the signal provides a faithful measure of import.

The amplitude of import extracted from the dose–response curve of CytB2_Δ43–65_-pep86 was fitted to an equation for specific binding, yielding a low dissociation constant (*K*_d_) of 12.7 ± 1.4 nM. Furthermore, the import rates vary in a hyperbolic manner with respect to precursor concentration, suggesting a Michaelis–Menten relationship, shown in the linearized Hanes–Wolf plot (Fig. 2e). This confirms that CytB2_Δ43–65_-pep86 import is limited by the number of import sites.

### Exploring energy-dependency of the mitochondrial import system

**Fig. 3:**
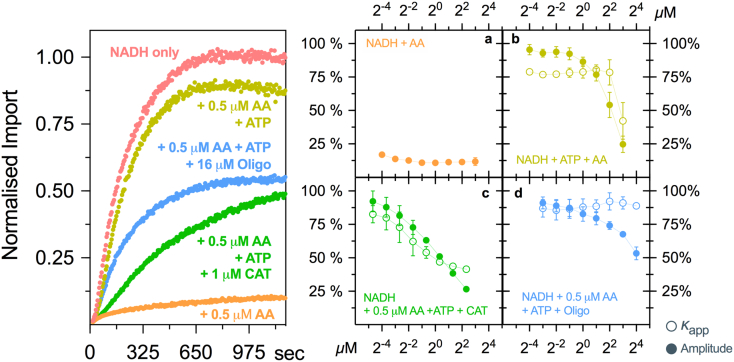
Energy-dependency of the mitochondrial import system. Mitochondria were energized in the presence of different mitochondrial poisons: AA (respiration inhibitior), Oligo (ATP synthase inhibitor) and/or CAT (adenine nucleotide translocase). Traces on the left represent typical import reactions in the presence of these drugs. Dose–response curves are presented in the graphs on the right; full circles represent amplitude of import, and empty circles show *k*_app_. Data are shown as mean ± SEM of two to three independent experiments. Error bars were omitted if smaller than symbols. Abbreviations: AA, antimycin A; CAT, carboxyatractyloside; Oligo, oligomycin.

Next, we explored the energy dependency of the mitochondrial import system, given that protein import is known to be ATP- and PMF-dependent (Fig. 3). When mitochondria were energized with NADH in the absence of ATP, the addition of the mitochondrial respiration inhibitor antimycin A (AA) was able to inhibit CytB2_Δ43–65_-pep86 import, regardless of the concentration used (Fig. 3a). This demonstrates the requirement of a functional respiratory chain to generate PMF and drive import. Under this condition, de-energized mitochondria can hydrolyse ATP in an attempt to generate ΔΨ. Therefore, addition of ATP as part of an ATP regenerating system was able to restore import, suggesting that the reverse activity of ATP synthase generates sufficient ΔΨ to drive CytB2_Δ43–65_-pep86 import (Fig. 3b). Interestingly, higher concentrations of AA in this setup caused additional inhibition of import (amplitude and rate), which we attributed to non-specific effects on mitochondrial physiology. Further evidence for the ATP synthase driving import in the presence of AA was obtained when matrix ATP influx was blocked by the adenine nucleotide translocase inhibitor carboxyatractyloside (CAT; Fig. 3c) or ATP hydrolysis by the ATP synthase inhibited with oligomycin (Oligo; Fig. 3d). In the first case, ATP uptake is blocked, and the effect is 2-fold—no ATP is available to drive import or to generate PMF. In the latter case, ATP enters the matrix but cannot be used to generate PMF due to inhibition by oligomycin. Although both drugs (CAT and Oligo) lowered the amplitude of import in a dose-dependent manner, oligomycin had no effect on *k*_app_ suggesting that ΔΨ determines the extent of CytB2_Δ43–65_ accumulation.

### Exploring the effect of small molecules on mitochondrial import

**Fig. 4:**
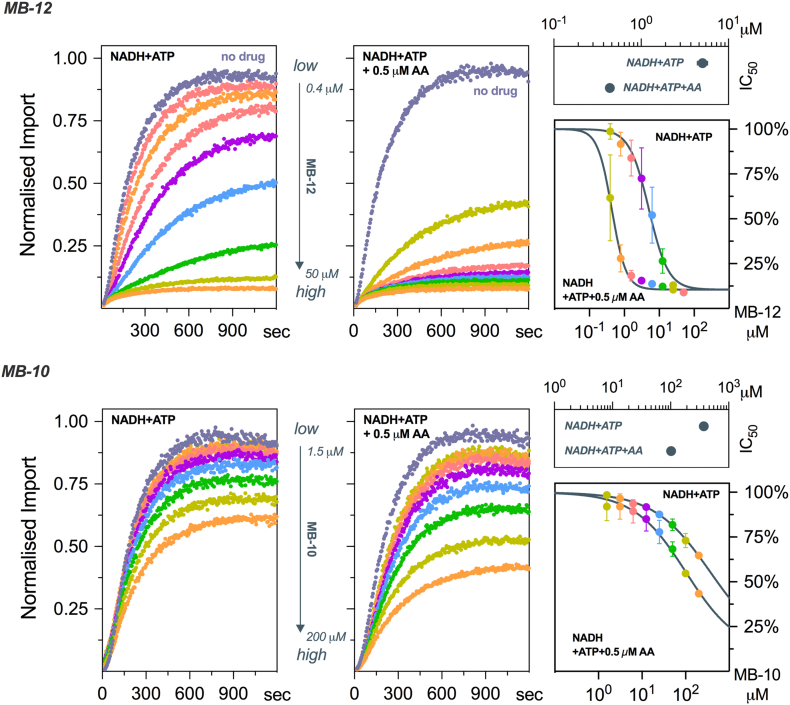
Effects of known inhibitors of mitochondrial import. Dose–response curves of MB-12 (top) or MB-10 (bottom) on the import of CytB2_Δ43–65_-pep86 into mitochondria in which PMF is created by NADH oxidation (left) or by the hydrolysis of ATP (right). Data are shown as mean ± SEM of three (MB-12) or two (MB-10) independent experiments, except for the IC50, where error bars represent 95% confidence intervals.

We used two small-molecule inhibitors of the TIM23 pathway, identified by the Koehler Lab, to validate the assay: MB-12, also known as dequalinium [4], and MB-10 [5]. In accordance with previous reports [4], we found that MB-12 causes a dose-dependent inhibition of CytB2_Δ43–65_-pep86 import with an IC_50_ 5.05 ± 0.32 μM (Fig. 4, top; “NADH + ATP”). The IC_50_ of MB-10, meanwhile, was 373 ± 16.4 μM (Fig. 4, bottom; “NADH + ATP”), significantly higher than previous reported [5]. Interestingly, both drugs showed a stronger inhibitory effect on translocation when the mitochondria were energized by the reverse activity of the ATP synthase, possibly reflecting non-specific effects (Fig. 4, “NADH + ATP + 0.5 μM AA”). This effect was more pronounced for MB-12 (IC_50_ = 0.437 ± 0.019 μM) than for MB-10 (IC_50_ = 102 ± 6.9 μM).

### Effect of signal sequence deletion

**Fig. 5:**
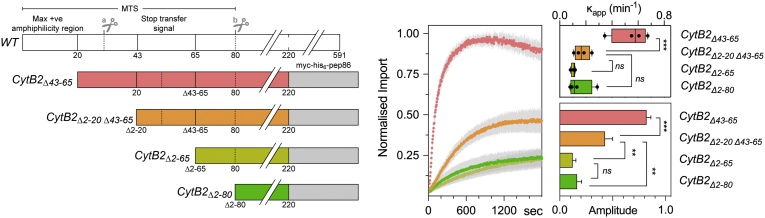
Effect of signal sequence deletion on mitochondrial import. The mitochondrial targeting sequence of CytB2_Δ43–65_-pep86 was truncated at different positions (diagram shown on the left-hand side with wild-type in white for comparison). Cutting points marked by “a” and “b” represent matrix and intermembrane space cleavage sites. Note that the “stop transfer signal” (shown in *WT*) is deleted in the other constructs. Purification tags and pep86 are depicted by the gray box. Import was measured at 1 μM precursor in fully energized mitochondria (NADH + ATP regeneration system). Average traces are shown in the middle plot, while secondary data are shown on the right-hand side. Data represent three to four independent experiments and are shown as mean ± SEM. Differences between groups were analyzed by a one-way ANOVA with predefined contrasts corrected with Holm–Sidak test. ***p < 0.001; **p < 0.01; ns, not significant.

Finally, we evaluated the effect of removing the presequences from the import substrates, anticipating that the mature part alone would not import. Previously, Klaus *et al*. [22] reported that deletion of the first 20 amino acids of CytB2 presequence was sufficient to render the precursor import-incompetent. However, because of the high sensitivity of our assay, we were able to observe that removal of the presequence (CytB2_Δ2–20 Δ43–65_-pep86) compromises but does not totally abolish import (Fig. 5). Careful inspection of CytB2_Δ2–20,43–65_-pep86 sequence using the MitoFates prediction algorithm (http://mitf.cbrc.jp/MitoFates/cgi-bin/top.cgi; access date: 30-05-2018) revealed that the construct still had > 70% probability to translocate to the matrix. Therefore, we deleted the remaining amino acids preceding the Δ43–65 truncation, yielding the CytB2_Δ2–65_-pep86, lacking both segments of its bipartite signal sequence. As expected, CytB2_Δ2–65_-pep86 was further compromised for import into mitochondria (Fig. 5). Deletion of the last 15 aa of CytB2 MTS (CytB2_Δ2–80_-pep86) showed impaired import to the same extent as the CytB2_Δ2–65_-pep86, suggesting that the first 43 aa comprise the major targeting signal to the matrix. This residual activity reveals a potentially interesting new feature of the precursor targeting determinants: one that, along with many other facets of the import process, can now be dissected in detail by the application of this validated new and powerful tool.

## Discussion

To understand protein transport across membranes, it is vital to be able to monitor it accurately and continuously. Until now, such experiments have been difficult to perform and scale and have yielded very little kinetic insight. The split-luciferase-based assay optimized and validated here solves these problems. Indeed, our success in adapting NanoBiT to different conditions, both in bacteria and in mitochondria, suggests that it will be compatible with many other membrane- and non-membrane-bound protein translocation machines.

Attempts to develop real-time import assays have been made previously, for example, by labeling pre-proteins with fluorescent dyes [23], [24]. These have provided some information on the mechanism of protein secretion by the bacterial Sec system; however, the risk of inefficient labeling represents possible competition for translocation by non-labeled protein, affecting overall kinetic analyses. And perhaps most importantly, fluorescent dyes are significantly different from amino acid side chains in terms of both size and chemical composition, raising questions as to the physiological relevance of the measurements. The pep86 tag circumvents these problems: it can be cloned easily onto the C-terminus of a pre-protein, and as a short peptide, it resembles exactly a native translocation substrate. Its relatively small footprint should not affect transport kinetics appreciably. Although we decided to place pep86 on the C-terminus of pre-proteins, the tag will bind with high affinity to 11S as long as it is available and not sterically hindered, which means that it could also be placed in internal protein loops, as previously reported [25].

Bipartite systems, such as the split-NanoLuc used in present work, have been used to evaluate protein translocation before [26], [27], [28]. Ensembled [29] or split-GFP [27] are limited by the fact that maturation of the chromophore (or that of any fluorescent protein) is a slow process, in the order of minutes [30]. This is much slower than protein translocation and therefore would be rate limiting, precluding any kinetic assessment of the biological process, and probably explains why it has been only really used for low time- and spatial-resolution *in vivo* analysis of protein localization. Split-β-galactosidase systems, such as CAPT [26] or PathHunter [28] (trademark of DiscoverX) are closer to our split-NanoLuc-based assay in that both rely on inactive fragments to restore enzyme activity. However, while Wehrman *et al*. [26] reported a tag of 46 aa in the CAPT system that spontaneously associates with a bigger fragment, the authors did not measure binding affinity, so the response time may be limited by the association of the fragments.

A possible disadvantage when using high 11S concentrations is fast depletion of its substrate furimazine. However, we lowered the amount of biological sample to avoid this problem while maintaining high sensitivity. It also suggests that in special cases where the amount of starting material for cell fractioning is an issue, such as mammalian cells, zebrafish, fruit flies or flatworms, our assay can be a practical tool to use.

The real-time assay showed a robust, reliable and high-dynamic range whenever it was challenged with standard controls for studying protein translocation. The signal readout, that is, translocation was energy-dependent and signal-sequence specific, and it could be unambiguously inhibited with known inhibitors of the translocon. An important aspect of the system is its ability to distinguish nuances beyond the capabilities of the classical methods; either in terms of signal sequences or to detect protein import in response to (patho)physiological energization conditions. For example, to our knowledge, it is the first time that protein import driven by the reversal of ATP synthase has been observed. In a cellular context, it means that respiration-impaired mitochondria can still efficiently import proteins as long as cytosolic ATP is available.

Contrarily to some recently developed high-sensitivity methods [31], [32], [33], [34] for measuring translocation (Table S1), the requirements for our new assays are a simple luminometer and standard molecular cloning procedures. Overall, this means that the real-time assay can be easily adopted by current or new laboratories working on protein translocation without the need of advanced knowledge on specific tools or techniques, while still providing high sensitivity and specificity.

The flexibility of the new assay shows that it is not restricted to the systems presented here but can be readily adapted to other frameworks, such as peroxisomes, chloroplasts, endoplasmic reticulum, nucleus or plasma membrane, or even for monitoring protein translocation in non-membrane-associated systems, such as to the interior of protein cages like GroEL or the proteasome.

Monitoring protein secretion through the plasma membrane using NanoBiT has already been described in mammalian cells [35] and in gram-positive bacteria [36]. However, employing the same approach to gram-negative bacteria is considerably challenging because the outer membrane in their cell envelope provides a low-permeability barrier. Therefore, instead of exogenous addition of 11S, we targeted it to the periplasm while co-expressing the “*dark*” peptide in the cytosol. This approach is preferable to the use of low-affinity tags/peptides [9], [26] because it ensures that spontaneous binding in the destination compartment is retained and paves the way to monitor β-lactamases secretion *in vivo* and in real-time. Thus, we believe that the assay will prove valuable for both fundamental research in protein trafficking systems and high-throughput drug screening.

## Material and Methods

A detailed description of the material and methods used can be found in the Online Supplement and they are described in outline below.

### Cloning

The genes were optimized for *E. coli* or *S. cerevisiae* according to their final use and purchased either as gene strings or as genes in plasmid. Then, they were cloned into the desired plasmid by restriction digestion or overlapping PCR. Plasmid DNA was amplified in α-select cells, while BL21(DE3) was used for protein expression instead.

### Protein expression

Cells harboring the desired expression plasmid were grown in 2xYT at 37 °C and induced with either arabinose or IPTG, depending on the plasmid, for 2.5–3 h. Generally, proteins were purified from inclusion bodies by IMAC followed by IEC. For details about each protein purification, please see the Full Methods in Supplemental Material.

### PLs and IMV preparation

SecYEG PLs and inverted membrane vesicles were prepared from the membranes as described previously [2]. IMVs were prepared from either *E. coli* BL21(DE3) or a strain lacking ATP synthase (unc-; HB1 cells).

### Mitochondrial isolation

Yeast cells expressing mt-11S were grown in YPG overnight, and then mitochondria were isolated through differential centrifugation after the cell wall was reduced and digested with zymolyase. Please see the Full Methods in Supplemental Material.

### Real-time import assay

In cuvette mode and for the Sec-system, the reaction mix was assembled in a 1-mL cuvette with a stirrer bar by adding the following (in order): TKM to give a final volume of 1 mL, Prionex (Sigma-Aldrich; registered trademark of Pentapharm AG, Basel) to 0.1%, 10 μL Nano-Glo substrate (furimazine; Promega), creatine phosphate to 5 mM, creatine kinase to 0.1 mg/mL, GST-dark to 40 μM, 1 μL SecYEG/11S HB1(DE3) IMVs or PLs, and SecA to 1 μM. After a 5-min equilibration, a luminescence baseline signal was measured for 1 min, followed by the addition of proOmpA-pep86 to 1 μM final concentration. After a further 10 min, ATP was added to 1 mM final concentration, and the transport reaction followed until completion. The cuvette was read on a Jobin Yvon Fluorolog (Horiba) with the lamp turned off and emission measured at 460 nm (with slits open to maximum, i.e., 10-nm bandpass).

In plate reader mode and for the mitochondrial system, reactions were carried out in 300 mM mannitol, 10 mM Hepes (pH 7.4), 25 μM EGTA, 1 mM KH_2_PO_4_ supplemented with 0.1% Prionex, 10 μM GST-dark, 2 mM NADH, 25–50 μg/mL of frozen yeast mitochondria and 0.25 × Nano-Glo substrate (furimazine; Promega), at 25 °C in a low-binding white 96-well plate. Creatine kinase (0.1 mg/mL), creatine phosphate (5 mM) and ATP (1 mM) were also included in the buffer unless stated otherwise. Reactions were started by the addition of 25 μL of precursor to make a final volume of 125 μL. Plates were read on a BioTek Synergy Neo2 plate reader (BioTek Instruments, UK) for 0.2 s/well, without emission filters, and the gain was set to allow for maximum sensitivity without detector saturation.

### Statistical analysis

Apparent rates (*k*_app_) were calculated as the reciprocal of the time it takes to reach half of the maximal luminescent signal (*t*_50%_). Statistical analyses were performed using GraphPad Prism version 8.0.0 (GraphPad Software, Inc., San Diego, CA, USA).

## Appendix A Supplementary data

Supplementary material enclosing full methods description, supplemental tables and figures. Image 1
