# Supplementary material for: A High-Resolution Luminescent Assay for Rapid and Continuous Monitoring of Protein Translocation across Biological Membranes
Source: J Mol Biol. 2019 Apr 5;431(8):1689–99. doi: 10.1016/j.jmb.2019.03.007 (PMC6461198; doi:10.1016/j.jmb.2019.03.007)
Supplement: Supplementary file 1 — Supplementary material enclosing full methods description, supplemental tables and figures. [file mmc1.pdf]

A HIGH-RESOLUTION LUMINESCENT ASSAY FOR RAPID AND CONTINUOUS MONITORING OF PROTEIN TRANSLOCATION ACROSS BIOLOGICAL MEMBRANES

Gonçalo C. Pereira[a], William J. Allen[a], Dan W. Watkins[a], Lisa Buddrus[a,b], Dylan Noone[a], Xia Liu[a], Andrew Richardson[a], Agnieszka Chacinska[c], Ian Collinson[a,b]

[a]*School of Biochemistry, Biomedical Sciences Building, University of Bristol, Bristol, UK*
[b]*BrisSynBio, University of Bristol, UK*
[c]*Centre of New Technologies, University of Warsaw, S. Banacha 2c, 02-097, Warsaw, Poland*

Abstract

Protein translocation is a fundamental process in biology. Major gaps in our understanding of this process arises due the poor sensitivity, low time-resolution and irreproducibility of translocation assays. To address this, we applied NanoLuc split-luciferase to produce a new strategy for measuring protein transport. The system reduces the timescale of data collection from days to minutes, and allows continuous acquisition with a time-resolution in the order of seconds – yielding kinetics parameters suitable for mechanistic elucidation and mathematical fitting. To demonstrate its versatility, we implemented and validated the assay in vitro and in vivo for the bacterial Sec system, and the mitochondrial protein import apparatus. Overall, this technology represents a major step forward, providing a powerful new tool for fundamental mechanistic enquiry of protein translocation and for inhibitor (drug) screening, with an intensity and rigour unattainable through classical methods.

Supplemental Material

Contents

1 Full Methods **3**
- 1.1 Reagents . . . 3
- 1.2 Culture conditions . . . 3
- 1.3 Cloning . . . 3
  - 1.3.1 mt-11S for mitochondrial preparations . . . 3
  - 1.3.2 His-tagged 11S for proteoliposomes preparations . . . 3
  - 1.3.3 Tethered 11S for IMV preparations . . . 4
  - 1.3.4 GST-dark peptide for in-vitro experiments . . . 4
  - 1.3.5 proOmpA(±pep86) . . . 4
  - 1.3.6 proSpy(±pep86) and mSpy(±pep86) . . . 4
  - 1.3.7 CytB2±pep86 and Δmts-CytB2-pep86 . . . 4
  - 1.3.8 pro11S–GST-dark for in-vivo experiments . . . 5
  - 1.3.9 NDM1-pep86 for in-vivo experiments . . . 5
- 1.4 Protein expression and purification . . . 5
  - 1.4.1 His-tagged 11S . . . 6

1.4.2 GST-dark peptide . . . 6
1.4.3 pep86-tagged mitochondrial precursors . . . 6
1.4.4 Pep86-tagged bacterial pre-proteins . . . 6
1.5 Proteoliposome (PL) preparation . . . 7
1.6 IMV preparation . . . 7
1.7 Mitochondrial isolation . . . 7
1.8 Binding experiments . . . 7
1.9 Western blot transport assays . . . 8
1.10 Real-time import assay . . . 8
1.10.1 Cuvette mode . . . 8
1.10.2 Microplate mode . . . 9
1.11 *In-vivo* β-lactamase secretion assay . . . 9
1.12 Data analysis and Statistics . . . 9

2 Supplemental Tables & Figures **10**

References **26**

List of Figures

S1 Experimental Design . . . 11
S2 Background signal on the bacterial systems – proteoliposomes . . . 12
S3 Effects of 11S concentration on pre-protein import traces . . . 12
S4 NDM1 translocation *in vivo*. . . . 13
S5 11S detection and determination on isolated mitochondria . . . 14
S6 Inhibitory effect of Furimazine on mitochondrial import. . . . 15
S7 Distinction between import and background traces. . . . 15
S8 Background signal in mitochondrial preparation . . . 16
S9 Kinetics analysis of precursor-pep86 association to 11S . . . 17

List of Tables

S1 New-generation methods for monitoring protein localization/translocation. . . . 10
S2 Effects of mitochondrial poisons on split-NanoLuc signal. . . . 10
S3 List of bacterial and yeast strains used in the current work. . . . 11
S4 List of plasmids used in the current work. . . . 13
S5 Sequences of constructs used in this current study. . . . 18

1. Full Methods

1.1. Reagents

All chemicals, such as antibiotics, inducers and mitochondrial poisons, were of the highest commercially available grade of purity and were purchased from Sigma-Aldrich. Aqueous solutions were prepared in ultrapure (type I) water (Milli-Q Biocel A10 with pre-treatment via Elix 5, Millipore, Billerica, MA, USA). For non-aqueous solutions, ethanol (99.5%) or dimetylsulfoxide (DMSO), both from Sigma-Aldrich, were used as solvent.

1.2. Culture conditions

Bacterial strains were cultured in LB or 2XYT for *in vivo* and *in vitro* experiments, respectively, with appropriate antibiotics (100 µg/mL ampicillin, 34 µg/mL chloramphenicol, 50 µg/mL kanamycin). Standard culturing temperature for *E. coli* MM52 (contains a temperature-sensitive genomic copy of SecA) was 30 °C and 37 °C was used for all other strains. Competent cells were prepared and transformed by heat-shock through standard procedures [1], with a 30 s incubation at 42 °C and recovery in LB only.

Wild-type yeast, Saccharomyces cerevisiae strain YPH499, were cultured in standard YPD (1% yeast extract, 2% peptone, 2% glucose) at 30 °C. Yeast mutants were cultured in synthetic complete growth media lacking uracil and supplemented with 2% glucose at 30 °C. For liquid cultures, media was further supplemented with 0.0025% penicillin and 0.0025% streptomycin. Competent cells were prepared as previously described [2] and transformed by the LiAc/PEG method. Briefly, competent cells were rinsed in water and incubated in transformation mix containing 0.5 ug DNA, 3 µg/mL salmon sperm DNA (Sigma-Aldrich, UK), 100 mM LiAc, 10 mM Tris pH 7.5, 1 mM EDTA and 40% PEG 3000, for 30 min at 30 °C followed by 15 min at 42 °C. Cells were let to recover in YPD for 60 min at 30 °C before plating on synthetic growth media lacking uracil (Kaiser mixture; Formedium, UK) and supplemented with 2% glucose.

1.3. Cloning

1.3.1. mt-11S for mitochondrial preparations

To produce yeast expressing 11S in the mitochondrial matrix, the 11S amino acid sequence previously published [3] was codon-optimised for S. cerevisiae and supplemented with the mitochondrial signal sequence of yeast alpha subunit of ATP synthase (ATP1/YBL099W; 1-35aa) on its N-terminus. This gene was purchased on a plasmid from Eurofins (Germany), digested with HindIII and XbaI, and ligated into the corresponding sites of pYES2CT (yielding pYES—mt-11S). The plasmid was verified by sequencing then transformed into YPH499 yeast cells. The mt-11S gene was cloned into a high-copy number plasmid (pYES2) under the control of GAL promoter to facilitate the delivery of high quantities of 11S to the mitochondrial matrix.

1.3.2. His-tagged 11S for proteoliposomes preparations

To produce and purify 11S, the 11S gene (without the mitochondrial signal sequence) was amplified from pYES—mt-11S with a 5' primer containing an NcoI site followed by a 6-his tag (GATCGTCCATGGGCCATCATCATCAT-CATCATGGCGTTTTCACATTGGAG), and a 3' primer containing a HindIII restriction site (GCCTAAAAGCTTC-TAGCTATTGATGGTTACACG). The resulting PCR product was digested with NcoI and HindIII, and ligated into the corresponding sites of pBAD/Myc-His C (yielding pBAD—$_{6H}$11S). The plasmid was verified by sequencing then transformed into BL21(DE3) cells.

1.3.3. Tethered 11S for IMV preparations

To produce IMVs with high concentrations of 11S on the inside, we tethered 11S to the periplasmic face of the inner membrane using a lipid anchor [4]. The *11S* gene (without the his-tag) was amplified from pBAD—$_{6H}$11S using a 5' primer with a NcoI restriction site followed by the signal sequence and first six amino acids of NlpA (EG10657; ACGTAGCCATGGGCAAACTGACAACACATCATCTACGGACAGGGGCCGCATTATTGCTG GCCGGAATTCT-GCTGGCAGGTTGCGACCAGAGTAGCAGCGGCGTTTTCACATTGGAG), and a 3' primer including the HindIII restriction site (CTACGTAAGCTTCTAGCT). The PCR product was then digested with NcoI and HindIII, and ligated into the corresponding sites in pRSFDuet-1. The resulting plasmid was verified by sequencing then co-transformed with pBAD—SecYEG into BL21(DE3) cells.

1.3.4. GST-dark peptide for in-vitro experiments

To allow recombinant expression and purification of large quantities of dark peptide, we fused it to the C-terminus of glutathione-S-transferase (GST). This was done by PCR insertion of a DNA sequence coding for a pep86 version that does not luminesce (dark peptide, see details in [5]) immediately after the BamHI site in pGEX-1 (primer sequences: CATCCTCCAAAATCGGATCCCGGAGTGAGCGGCTG GGCGCTGTTTAAAAAAATTAGC-TAAGAATTCATCGTGACTGAC and GTCAGTCACGATGAATTCTTAGCTAATTTTTTTAAACAGCGCCCAGC-CGCTCACTCCGGGATCCGATTTTGGAGGATG). The resulting plasmid (pGEX—GST-dark) was verified by DNA sequencing, then transformed into BL21 (DE3) cells.

1.3.5. proOmpA(±pep86)

proOmpA with a C-terminal minimal V5 epitope was produced as described previously [6]. For the real-time translocation assays, PCR insertion was used to add the pep86 sequence, preceded by a short GSG linker, after the V5 tag (primers: GAATCCGCTGCTGGGCCTGGGCTCCGGCGTGAGCGGCTG GCGCCTGTTTAAAAAAATTAGC-TAAGCTTACGTAGAACAAAAAC and GTTTTTGTTCTACGTAAGCTTAGCTAATTTTTTTAAACAGGCGCCAGC-CGCTCACGCCGGAGCCCAGGCCCAGCAGCGGATTC). After verifying the clone by DNA sequencing, proOmpA-pep86 was expressed and purified using exactly the same protocol as standard proOmpA.

1.3.6. proSpy(±pep86) and mSpy(±pep86)

To produce the periplasmic chaperone proSpy (EG13490), an *E. coli* optimised gene string (Life Technologies) for proSpy with a C-terminal minimal V5 epitope, a linker with pep86 tag, a TEV cleavage sequence and a 6-his tag was cloned into pBAD-HisA using overlap extension mutagenesis. For the mature form of Spy, a separate gene string was purchased omitting the signal sequence (1-23 aa). The resulting plasmids, pBAD—proSpy-pep86 and pBAD—mSpy-pep86, were verified by DNA sequencing, then transformed into MM52 and BL21(DE3), respectively.

1.3.7. CytB2±pep86 and Δmts-CytB2-pep86

An engineered version of yeast cytochrome B2 (YML054C, [7]) comprising the first 158 aa with its hydrophobic domain on the signal sequence deleted (Δ43-65) followed by two tandem TEV cleave sites, a Myc tag and a C-term 6xhis tag, was codon-optimised for *E. coli* and purchased as a gene on plasmid (Eurofins, Germany). Then, the plasmid was digested with NcoI and HindIII, and the insert ligated into the corresponding sites of pBAD/Myc-His C (yielding pBAD–CytB2$_{\Delta 43\text{-}65}$). For the real-time translocation assays, PCR insertion was used to add the pep86 sequence, preceded by a SGGGGS linker, after the 6xhis tag, yielding pBAD–CytB2$_{\Delta 43\text{-}65}$-pep86. All plasmids were verified by sequencing then transformed into BL21(DE3) cells.

For CytB2 with different truncations on its mitochondrial targeting sequence, an *E. coli* optimised sequence of CytB2 comprising the first 220 aa and the Δ43-65 deletion was supplemented with a Myc tag, a 6xhis tag followed by a GGGS linker and a C-term pep86 tag. The sequence was purchased as gene on plasmid (Eurofins) and digested with NcoI and HindIII. Then, the insert was ligated into the corresponding sites of pBAD/Myc-His C (yielding pBAD–$CytB2^{220}_{\Delta 43-65}$-pep86). The different MTS truncations were achieved by plasmid PCR deletion using pBAD–$CytB2^{220}_{\Delta 43-65}$-pep86 as template and the following primers to yield the corresponding plasmids: GCGAGCAAAACCCGGTTAAATAC and CATGGTTAATTCCTCCTGTTAGCC for pBAD–$CytB2^{220}_{\Delta 2-20\ \Delta 43-65}$-pep86; TCTAGTGTTGCGTATCTGAATTGG and CATGGTTAATTCCTCCTGTTAGCC for pBAD–$CytB2^{220}_{\Delta 2-65}$-pep86, and; GAACCGAAACTCGACATGAACAAA and CATGGTTAATTCCTCCTGTTAGCC for pBAD–$CytB2^{220}_{\Delta 2-80}$-pep86. All plasmids were verified by sequencing then transformed into BL21(DE3) cells.

1.3.8. pro11S–GST-dark for in-vivo experiments

To produce bacteria expressing 11S in the periplasm, the amino acid sequence of 11S was codon-optimised for *E. coli*, supplemented with the signal sequence of bacteria OmpA (EG10669, 1-21 aa) on its N-terminus (pro11S) and purchased as a gene fragment (GeneArt, Invitrogen). The fragment was ligated into pMiniT2.0 according to the manufacturer's instructions (NEB PCR cloning kit, E1203S), and sequence checked with pMiniT F and R primers. pMini—pro11S and pBAD-Myc-His-C were digested with NcoI and HindIII (NEB HF enzymes) according to the manual, fragments agarose gel purified (QIAquick gel extraction kit, #28704), ligated using T4 ligase (ThermoScientific, #EL0011) and transformed into α-select creating pBAD—pro11S.

The GST-dark gene fragment was synthesised (Invitrogen), ligated into pMiniT2.0 according to the manual and sequence checked. pMini—GST-dark and pBAD—pro11S were digested with HindIII and SalI (NEB HF enzymes) according to the manual, fragments agarose gel purified, ligated using T4 ligase and transformed into *E. coli* α-select creating pBAD—pro11S—GST-dark.

1.3.9. NDM1-pep86 for in-vivo experiments

The pep86 tag was added to the 3' end of *ndm-1* by site-directed mutagenesis PCR (Phusion Site-Directed Mutagenesis Kit, ThermoScientific, #F541) using 5' phosphorylated primers (forward: 5' PHO-CTGTTTAAAAAAATTAGCT AAGCCATGGCTGACCACGTCACC and reverse: 5' PHO-GCGCCAGCCGCTCACGCCGCTGCCGCGCAGCTTG TCGGC) according to manual (64°C annealing temperature for 30 s, 90 s extension) and pSU2718-NDM1 as template. The circularised plasmid was ligated according to manufacturer's instructions and transformed into *E. coli* α-select creating pSU2718-NDM1-pep86.

1.4. Protein expression and purification

For expression, pre-cultures were inoculated with a single colony of the desired bacterial strain (BL21(DE3) as default) and grown in LB with appropriate antibiotic for 16 h at 37 °C, 200 rpm. Cultures were inoculated at 1:100 from pre-cultures in 2xYT plus antibiotic and grown at 37 °C, 200 rpm until mid-log phase, then induced for 2.5-3 h with 0.1-0.2% (w/v) arabinose or 1 mM depending on the plasmid. For overexpression of bacterial pre-proteins the MM52 strain was used instead as it contains a temperature-sensitive copy of genomic SecA [8]. When exposed to temperatures above 30 °C the mutant SecA is rendered inactive, causing pre-proteins to accumulate in the cytoplasm, typically as inclusion bodies. Therefore, pre-cultures were grown at 30°C and protein expression carried out at 39 °C.

1.4.1. His-tagged 11S

Cells were harvested, resuspended in 20 mM Tris pH 7.5, 50 mM KCl (TK) with 10% glycerol (TKG) then cracked open using a cell disruptor (Constant Systems) and clarified by centrifugation. The supernatant was loaded onto a Ni^{2+} column packed with chelating Sepharose Fast Flow resin (GE Healthcare), washed in TKG with 50 mM imidazole, then eluted with TKG + 330 mM imidazole. Imidazole was removed by washing with TKG in a spin concentrator, and the final protein concentration determined from A_{280}, using the calculated extinction coefficient of 19,940 $M^{-1}.cm^{-1}$. The sample was then snap frozen and stored at -80 °C.

1.4.2. GST-dark peptide

Cells were harvested, resuspended in TK buffer then cracked in a cell disruptor and clarified by centrifugation. The supernatant was loaded onto a GSTrap 4B (GE Healthcare) at 4 °C and the column washed with TK buffer until A_{280} of the flowthrough stopped decreasing. Elution was performed with 10 μM reduced glutathione in TK. The yield of the resulting protein (hereafter GST-dark) was determined from A_{280}, using the calculated molar extinction coefficient of 48,360 $M^{-1}.cm^{-1}$. The sample was then snap frozen and stored at -80 °C.

1.4.3. pep86-tagged mitochondrial precursors

Cells were harvested, resuspended in TK buffer then cracked in a cell disruptor and clarified by centrifugation. Inclusion bodies were solubilised in TK plus 6 M urea (TK + urea) before loading into an in-house packed Ni^{2+} column. After washing with TK + urea, proteins were eluted with 330 mM imidazole in TK + urea and loaded into an in-house packed Q- or S-column. After column wash, proteins were thereafter eluted in TK + urea + 1 M KCl gradient (0-100%) during 20 min. Final fraction was spin concentrated and the final protein concentration determined from A_{280}, using the calculated extinction coefficient. The sample was then snap frozen and stored at -80 °C.

1.4.4. Pep86-tagged bacterial pre-proteins

For **proOmpA**, the cell pellet was resuspended in 130 mM NaCl, 20 mM Tris pH 8.0 and cells cracked in a cell disruptor followed by a clarifying spin. A previously established purification protocol was utilised where inclusion bodies were harvested by gentle centrifugation at 4000 g for 15 min and solubilised in urea [6]. The resulting mixture was loaded onto an anion exchange column equilibrated in a salt-free 6 M urea, 10 mM Tris pH 8.0 buffer. A linear salt gradient of 0-1 M was then applied, where proOmpA-pep86 constituted the first protein to elute, with a 280 nm absorbance peak at approximately 40 mM NaCl.

For **proSpy**, cells were harvested and resuspended in lysis buffer (500 mM NaCl, 50 mM Tris, 30 mM imidazole, pH 8.0) buffer supplemented with cOmplete, EDTA-free Protease Inhibitor Cocktail. The cells were then lysed and clarified by centrifugation. The soluble cell fraction was determined to contain approximately 80% of the total expressed Spy and was therefore loaded onto a 5 mL HiTrap Crude FF nickel affinity chromatography column (GE Healthcare). After washing with lysis buffer, the bound proteins were eluted with lysis buffer containing 300 mM imidazole and then spin-concentrated (5 KDa cut-off) to ~14 mL before TEV digestion. DTT and EDTA, 1 mM and 0.5 mM respectively, plus ~0.1 mg/mL TEV protease were added to the suspension and incubated at room temperature for about 3 h. Then, Spy solution was purified by nickel affinity chromatography as described above, but this time collecting the unbound column flow through (His tag removed-Spy). The sample was dialysed overnight into 6 M urea, 20 mM Tris pH 8.0 and then snap frozen and stored at -80 °C.

1.5. Proteoliposome (PL) preparation

SecYEG proteoliposomes were produced as described previously [6]. Briefly, purified SecYEG was mixed with *E. coli* polar lipids in DDM, then the detergent removed gradually using BioBeads. To encapsulate 11S, we simply included purified 11S at the desired final concentration (20 μM standard, or as noted in the text) in the SecYEG/polar lipid mix, prior to the addition of biobeads. For the initial experiments, SecYEG/11S PLs were harvested by centrifugation (30 min at 100,000 g) as per the standard method, washed twice by resuspending in 3 mL TKM then centrifuging for 30 min at 100,000 g and pipetting off the supernatant, then resuspended to give the desired final SecYEG concentration. These additional washing steps removed most of the non-encapsulated 11S, reducing the background signal for the transport assays – although they did not obviate the need for GST-dark.

For the 11S concentration series we instead passed the reconstituted PLs over a gravity flow Sephacryl-S1000 column to separate away unbound 11S. These PLs were quantified by scattering at 320 nm (relative to PLs produced using the standard method), then used directly in transport assays. This method is both more effective at removing free 11S and eliminates the centrifugation and resuspension steps, which potentially damage PLs and cause them to leak.

1.6. IMV preparation

IMVs were either prepared from *E. coli* BL21(DE3) or a strain lacking ATP synthase (unc-; HB1 cells [9]), to prevent PMF generation upon addition of ATP. Cells were grown to mid-log phase and 37 °C in 2xYT supplemented with 100 μg/mL ampicillin and 50 μg/L kanamycin, then co-induced for 2.5 h with 0.1% arabinose and 1 mM IPTG. Inverted membrane vesicles were prepared from the membranes as described previously [6].

1.7. Mitochondrial isolation

mt-11S-expressing yeast were grown overnight at 30 °C in synthetic growth media lacking uracil and supplemented with 3% glycerol plus 0.0025% Pen/Strep. Yeast cells were cultured in glycerol-based media to increase mitochondrial mass and maximise mitochondrial function [10] 1% galactose was added at mid-log phase to start inducing mt-11S (total time ~16h). In the end, mitochondria were isolated through differential centrifugation after cell wall was reduced by 1 mM DTT in 100 mM Tris-SO4 buffer for 15 min at 30 °C and then digested with zymolase in sorbitol-phosphate buffer for 30 min at 30 °C. The final mitochondrial pellet was resuspended in 250 mM sucrose and 10 mM MOPS, pH 7.2. Mitochondrial protein was quantified by BCA assay, using BSA as standard.

1.8. Binding experiments

Complementation of pep86-tagged precursor with pure $_{6H}$11S was performed in 1x Nano-Glo buffer diluted with TK buffer and Prionex (0.1% final). The reaction mix containing 1x furimazine was used to prepare a titration curve of precursor ranging from 13.9 nM to 3 μM which was added to a 96-well plate. Separately, pure 11S was diluted in reaction mix supplemented with 1x furimazine so that automatic injection of 80 μL would give the desired concentration upon addition (30 pM in 100 μL). Luminescence was read on BioTek Synergy Neo2 plate reader (BioTek Instruments, UK) without emission filters every 0.5 s during 30 s at 25 °C working on well mode. Obtained data was fitted to a single exponential.

1.9. Western blot transport assays

For the **Sec-system**, western blot transport time courses were performed in a 25 °C heat block. Reaction master mixes were prepared in buffer TK + 2 mM MgCl2 (TKM), containing: creatine phosphate to 5 mM, creatine kinase to 0.1 mg/mL, SecYEG IMVs or PLs to 4% of final volume, SecA to 1 µM and proOmpA to 0.2 µM. From each master mix, 5 µL was set aside for a 10% control, and another 50 µl diluted 5-fold into ice-cold 1 mg/mL protease K in 5 mM EDTA as a -ATP (t=0) control. Reactions were immediately started by adding ATP to a final concentration of 1 mM, and 50 µL was taken at various time points and quenched rapidly as for the t = 0 sample. Samples were prepared and western blotted essentially as described previously [6]. Briefly, undigested protein was precipitated on ice for 30 min with 20% (w/v final) trichloroacetic acid, then centrifuged and the supernatant removed. Pellets were dried in a vacuum centrifuge, then resuspended in 30 µL 2.5x NuPage LDS buffer (Thermo Fisher Scientific) and heated to 70 °C for 20 min. 10 µL of the resulting samples were run out on a gel, blotted, then developed using an α-v5 primary antibody (SV5-Pk1, GeneTex) and a DyLight 800-labelled secondary antibody (Thermo Fisher Scientific). Western blots were visualised on an Odyssey Fc (LI-COR) and the bands quantified using the built-in software.

For the **mitochondrial system**, yeast mitochondria (240 µg protein) were diluted in 240 µL import buffer (250 mM sucrose, 80 mM KCl, 5 mM MgCl2 , 10mM K2HPO4 , 10 mM MOPS-KOH pH 7.2) supplemented with 2 mM NADH, 2 mM ATP, 5 mM creatine phosphate and 0.1 mg/mL creatine kinase. The samples were preincubated at 25 °C for 5 min before import was started by adding 1 µg/mL substrate. The reaction was allowed to proceed for 1, 3 or 5 min with gentle shaking at 350 rpm before being stopped by the addition of 2.4 µL VOA (containing 100 µM valinomycin, 2 mM Oligomycin and 800 µM antimycin A). Half of each sample was treated with 25 µg/mL Proteinase K on ice for 15 min followed by the addition of 3 mM phenylmethylsulfonyl fluoride (PMSF) for 2-5 min to stop the reaction. Centrifugation at 20,000 g was used to isolate the mitochondria which were then washed with SM buffer (250 mM sucrose, 10 mM MOPS-KOH, pH7.2). Pellets were resolved in 2x sample buffer (4% SDS, 20% glycerol, 125 mM Tris-HCl pH 6.8, 0.02% bromophenol blue, 50 mM DTT) and heated at 65 °C for 10 min before being subjected to SDS-PAGE. After blotting, membranes were developed using an anti-myc primary antibody (Cell Signaling) and a DyLight 800-labelled secondary antibody (Thermo Fisher Scientific). Western blots were visualised on an Odyssey Fc (LI-COR) and the bands quantified using the built-in software.

1.10. Real-time import assay

1.10.1. Cuvette mode

Real-time import assays (for the Sec system) were performed at 25 °C in a Jobin Yvon Fluorolog (Horiba) with the lamp turned off and emission measured at 460 nm (with slits open to maximum, i.e. 10 nm bandpass). A reaction mix was assembled in a 1 mL cuvette with a stirrer bar by adding (in order): TKM to give a final volume of 1 mL, Prionex (Sigma-Aldrich; registered trademark of Pentapharm AG, Basel) to 0.1%, 10 µL Nano-Glo substrate (furimazine, Promega), creatine phosphate to 5 mM, creatine kinase to 0.1 mg/mL, GST-dark to 40 µM, 1 µL SecYEG/11S HB1(DE3) IMVs or PLs, and SecA to 1 µM. After a 5 min equilibration, a luminescence baseline signal was measured for 1 min, followed by addition of proOmpA-pep86 to 1 µM final concentration. After a further 10 min, ATP was added to 1 mM final concentration, and the transport reaction followed until completion.

The background, caused by association of proOmpA-pep86 with non-encapsulated 11S, fits well to a single (PLs) or double (IMVs) exponential (Fig. 2). Therefore, we fitted the signal from after proOmpA-pep86 but prior to ATP addition to a single exponential, and subtracted the resulting fit from the raw data. This corrected data corresponds to ATP-driven protein transport.

1.10.2. Microplate mode

For the **Sec-system**, all reactions were carried in 20 mM Tris, 50 mM KCl, 2 mM MgCl2, pH 8.0 (TKM) supplemented with 0.1% Prionex, 0.1 mg/mL creatine kinase, 5 mM creatine phosphate, 40 µM GST-dark, 1x Nano-Glo substrate (furimazine, Promega), 0.3 µM SecA and at 25 °C in a low-binding white 96 well plate. Reaction wells were setup with 3 µL of pre-protein (final concentration approximately 3 µM) and, immediately prior to starting measurements, 97 µL of master mix, such that on automated injection of 25 µL of 5 mM ATP solution the above concentrations were achieved. Background luminescence (likely caused by 11S from damaged IMVs) was measured for 10 min on a BioTek Synergy Neo2 plate reader (BioTek Instruments, UK) until equilibration was achieved (steady-state luminescence), at which point 25 µL of ATP was automatically injected and mixed thoroughly (for 2 s) to initiate translocation. Luminescence was collected without emission filters for an additional 20 min, with the lowest interval time possible for the number of samples being measured.

For the **mitochondrial system**, reactions were carried out in 300 mM mannitol, 10 mM HEPES pH 7.4, 25 µM EGTA, 1 mM KH2PO4 supplemented with 0.1% Prionex, 10 µM GST-dark, 2 mM NADH, 25-50 µg/mL of frozen yeast mitochondria and 0.25x Nano-Glo substrate (furimazine, Promega), at 25°C in a low-binding white 96 well plate. Creatine kinase (0.1 mg/mL), creatine phosphate (5 mM) and ATP (1 mM) were also included in the buffer unless stated otherwise. Reactions started by the addition of 25 µL of precursor to make a final volume of 125 µL.

When plates were read on a Packard Lumicount BL10001 (Packard BioSciences, Meriden, CT, US), additions were made manually using an 8-channel pipette with manual mixing; if BioTek Synergy Neo2 plate reader (BioTek Instruments, UK) was used instead, additions were made automatically using the system pump set to the default injection speed followed by a 5 s linear shaking step. In both plate readers, luminescence was collected for 0.2 s/well, without emission filters, and the gain was set to allow maximum sensitivity without detector saturation. An initial baseline of 60 sec was acquired before precursor addition and then luminescence was read for at least 20 min. Time between reads was set to the minimum allowed interval in each plate reader – 10-12 s for LumiCount and 5 s for Neo2.

1.11. In-vivo β-lactamase secretion assay

E. coli MC4100 were transformed with pBAD—pro11S–GST-dark alone or in combination with pSU2718–NDM-1-pep86. Starter cultures were inoculated with a single colony of the desired strain and grown in LB with appropriate antibiotic for 16 h at 30 °C, 200 rpm. 5 mL cultures (LB with antibiotic) were inoculated from these starter cultures at 1:100 and grown at 30 °C, 200 rpm until OD600∼1. At that point, all cultures were induced by adding arabinose to a final concentration of 0.2% (w/v) and incubated at 39 °C. 100 µL of sample was taken at the time of induction and assayed 2 h later using the Nano-Glo Live Cell Assay System (Promega) according to the manufacturer's protocol. Luminescence was measured over 100 reads during 10-20 min and the data averaged. Background luminescence was deducted from assay data of bla carrying strains.

1.12. Data analysis and Statistics

Results are shown as means±SEM of the indicated number of experiments. Apparent rates (k_{app}) were calculated as the reciprocal of the time it takes to reach half of the maximal luminescent signal ($t_{50\%}$). Statistical significance between mean differences was determined using two-tailed Student's t test or one-way ANOVA, when more than two groups were analysed, followed by predefined contrasts using Bonferoni's post-hoc analysis to correct for multiple comparisons. Differences were considered significant if p ¡ 0.05 and categorized accordingly to their interval of

confidence. Statistical analyses were performed using Graph Pad Prism version 8.0.0 (GraphPad Software, Inc., San Diego, CA, USA).

2. Supplemental Tables & Figures

Table S1: New-generation methods for monitoring protein localization/translocation.

Type	Name	Reporter	Readout	System	Cell Type	References
Complementation	CAPT	Split β-galactosidase	Chemiluminescence	*in vivo*	Mammalian	[11]
		Split-GFP	Fluorescence	*in vivo*	Mammalian	[12]
	PathHunter	β-galactosidase fragment	Chemiluminescence	*in vivo*	Mammalian	[13]
Redistribution		GFP-fused POI	Fluorescence	*in vivo*	Mammalian	[14]
		Fluorescent SNAP-tag labelled POI	Fluorescence (WB)	*in vitro*	Yeast mitochondria	[15]
Dye labelling		Protease protection of fluorescein-labelled POI	Fluorescence (WB)	*in vitro*	Bacterial IMVs	
		Dequencing of Atto565 Ni^{2+} pair	Fluorescence	*in vitro*	Bacterial IMVs	[16]
Bio-orthogonal reaction	RING-MITO	eGFP by protein splicing with inteins	Fluorescence	*in vivo*	Mammalian	[17][18]
	MITO-APEX	Peroxidase-dependent proximity biotinylation	Mass Spectometry	*in vivo*	Mammalian	[19]
	ClickIn	Click chemistry of mitochondria-targeted cyclooctyne	Mass Spectometry	*in vitro*	Rat liver mitochondria	[20]

Abbreviations: IMVs - inner membrane vesicles, POI - protein of interest, WB - western blot.
Disclaimer: The list above is not an exhaustive representation of all the methods available at the moment of publication, we would like to acknowledge all the colleagues who have developed assays but are not listed above.

Table S2: Effects of mitochondrial poisons on split-NanoLuc signal.

Compound	Target	Low concentration (0.1 μM)	High concentration (1 μM)
DMSO	no target (vehicle only)	100 %	100 %
Nigericin	ΔpH	97.67 %	107.42 %
Valinomycin	$\Delta\Psi$	90.70 %	96.90 %
Antimycin A	Complex III	102.17 %	97.86 %
Oligomycin	Complex V	89.41 %	103.59 %
CCCP	$\Delta\Psi$	89.13 %	**43.32 %**
DNP	$\Delta\Psi$	93.97 %	98.40 %

CytB2$_{\Delta 43\text{-}65}$-pep86 was mixed with pure $_{6H}$11S and furimazine in TK buffer pH 7.4. Data was fit to one-site binding curve and the RLU_{max} was used to compare between different drugs. n=1.

Table S3: List of bacterial and yeast strains used in the current work.

Organism	Strain	Use	Relevant genotype	Source
E. Coli	α-select	cloning strain	*F-, deoR, endA1, recA1, relA1, gyrA96, hsdR17(rk-, mk+), supE44, thi-1, phoA, Δ(lacZYA argF)U169, φ80lacZΔM15λ -*	Bioline & Lab stock
	BL21(DE3)	expression strain	*B, F-, ompT, gal, dcm, lon, hsdSB(rB–mB–), λ(DE3 [lacI lacUV5-T7p07, ind1, sam7, nin5]), [malB+]K-12(λS)*	Lab stock
	MC4100	expression strain	*F-, [araD139]B/r, Δ(argF-lac)169, λ-, e14-, flhD5301, Δ(fruK-yeiR)725(fruA25), relA1, rpsL150(strR), rbsR22, Δ(fimB-fimE)632(::IS1), deoC1*	Coli Genetic Stock Centre
	MM52	expression strain	*F-, [araD139]B/r, secA51(Ts), Δ(argF-lac)169, λ-, flb-5301, Δ(fruK-yeiR)725(fruA25), relA1, rpsL150(strR), rbsR22, Δ(fimB-fimE)632(::IS1), deoC1*	Coli Genetic Stock Centre
S. cerevisiae	YPH499	cloning strain	*MATa, ura3-52, lys2-801(Am), ade2-101(Oc), trp1-Δ63, his3-Δ200, leu2-Δ1*	Prof. Agnieszka Chacińska Lab

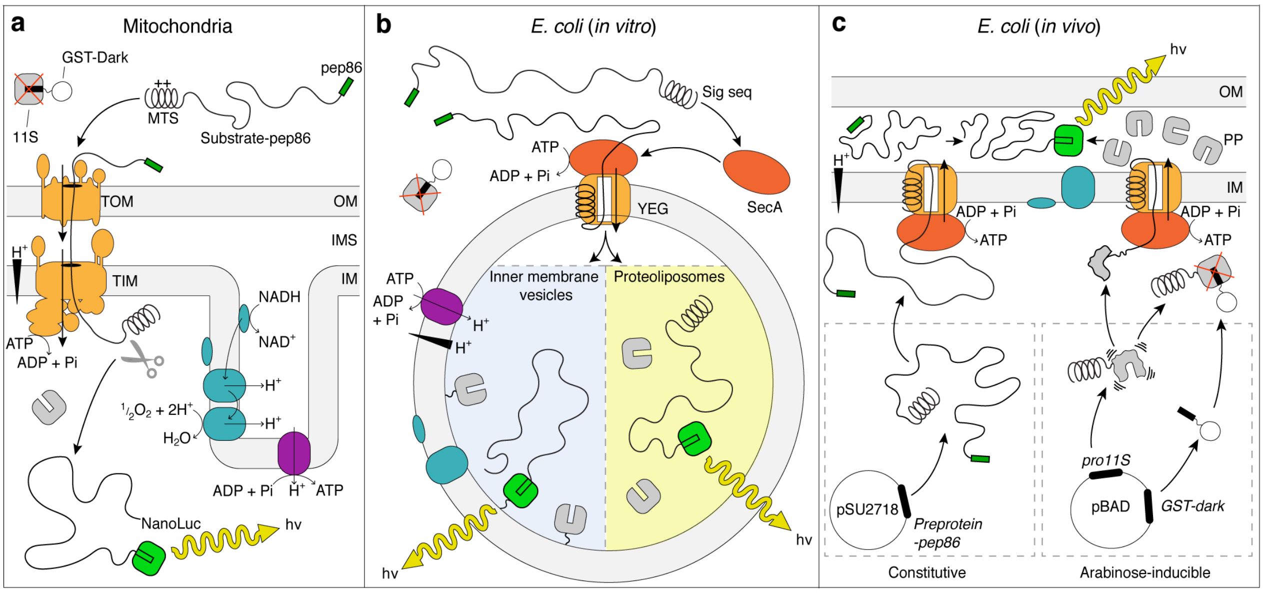

Figure S1: Overview of the new real-time assay to monitor protein translocation on different biological systems. The large 11S fragment was segregated in the mitochondrial matrix, proteoliposome lumen, tethered to the inner membrane of *E. coli* (inner membrane vesicles) or in the periplasm (*in vivo*). All pre-protein substrates were tagged on their C-terminus with the high-affinity peptide pep86. To decrease signal background, a non-luminescent high-affinity pep86 peptide (GST-dark) was added to the reactions (mitochondria, *E. coli in vitro*) or co-expressed in the cytosol for in vivo experiments. In all systems, successful pre-protein translocation was observed as an increase in luminescent signal upon pep86 \ 11S complementation. Abbreviations: hv – light; IM – inner membrane; IMS – intermembrane sequence; MTS – mitochondrial targeting sequence; OM – outer membrane; PP – periplasm; Sig. seq. – signal sequence; TIM – translocase of inner membrane; TOM – translocase of outer membrane.

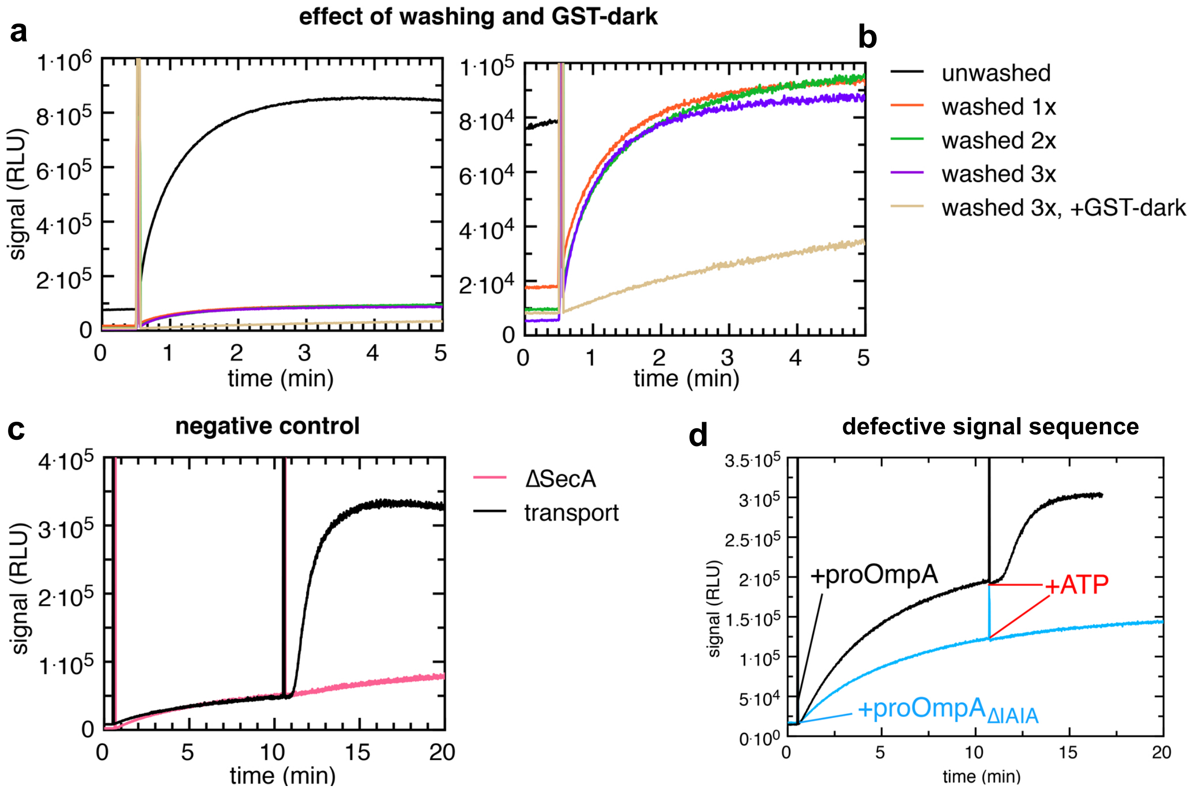

Figure S2: **a** – Effect of 'washing' PLs and background removal. Cycles of centrifugation and resuspension reduce the signal background, but do not remove it completely. Including *GST-dark* further decreases the background, but still does not fully obliviate it; **b** – zoomed version of 'a'; **c**, **d** – raw data for the negative control experiments in Fig. 1c, showing that both SecA and a functional signal sequence are required for import. n=1 for all panels.

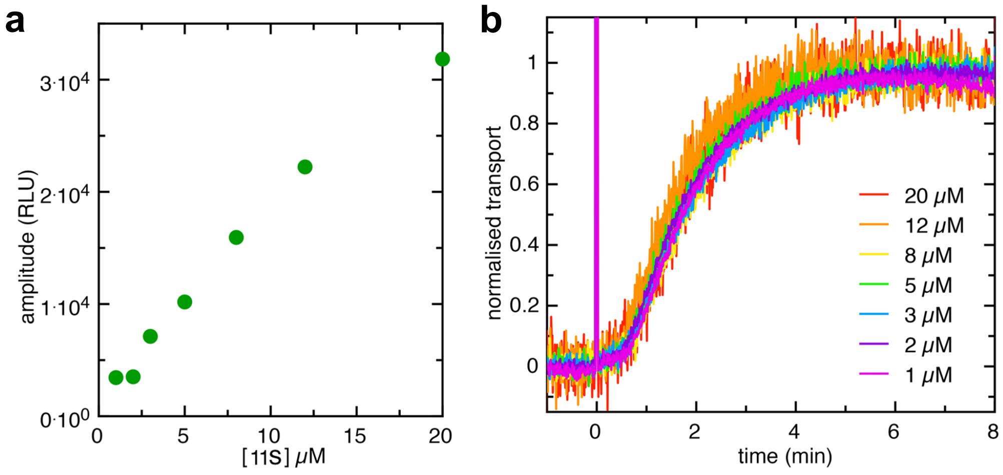

Figure S3: Effects of 11S concentration on pre-protein import traces. Despite the positive correlation between 11S concentration and luminescence signal (left-hand plot) the shape of the curve after normalisation to RLU_{max} remains the same (right-hand plot), confirming that the assay reports protein translocation rather than complementation of both fragments.

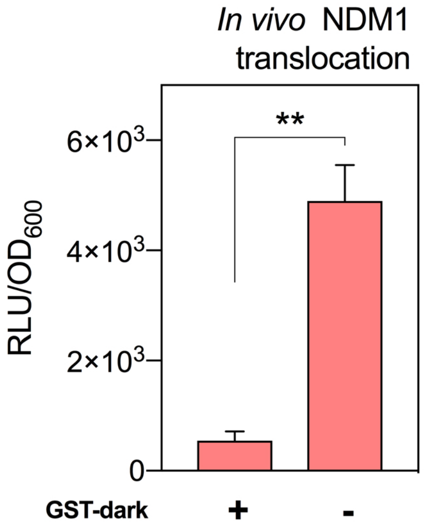

Figure S4: Effect of GST-dark co-expression with 11S for monitoring NDM1-pep86 translocation *in vivo* in MC4100 cells. Data is shown as mean ± SEM of three independent experiments. Differences between groups were assessed by Student's t-test.

Table S4: List of plasmids used in the current work.

Plasmid Name	Use	Relevant genotype	Source
pBAD	Cloning vector	*ara*C, P_{BAD}, *myc*, His, *rrnB*, Amp^R, *ori*pBR322	Lab stock
pBAD–$_{6H}$11S	Expression vector	pBAD carries *11S* gene with N-term 6xHis tag	This study
pBAD–NlpA-11S	Expression vector	pBAD carries *11S* gene with signal sequence of *NlpA*	This study
pBAD–proOmpA	Expression vector	pBAD carries *OmpA* gene	This study
pBAD–proOmpA-pep86	Expression vector	pBAD–pOmpA with C-term pep86 tag	This study
pBAD–proSpy	Expression vector	pBAD carries *Spy* gene	This study
pBAD–proSpy-pep86	Expression vector	pBAD–proSpy with C-term pep86 tag	This study
pBAD–mSpy	Expression vector	pBAD–proSpy with signal sequence deleted	This study
pBAD–mSpy-pep86	Expression vector	pBAD–proSpy-pep86 with signal sequence deleted	This study
pBAD–$CytB2_{\Delta 43\text{-}65}$	Expression vector	pBAD carries $\mathit{CytB2}_{\Delta 43\text{-}65}$ gene	This study
pBAD–$CytB2_{\Delta 43\text{-}65}$-pep86	Expression vector	pBAD-$CytB2_{\Delta 43\text{-}65}$ with C-term pep86 tag	This study
pBAD–pro-11S	Expression vector	pBAD carries *11S* gene with signal sequence of *OmpA*	This study
pBAD–pro-11S-GST-dark	Expression vector	pBAD–pro-11S carries *GST-dark* gene	This study
pGEX-1	Cloning vector	GST, P_{tac}, lacI^q, Amp^R, *ori*pBR322	Lab stock
pGEX–GST-dark	Expression vector	pGEX-1 carries non-luminescent pep86 fused to GST	This study
pSU2718-NDM-1	Cloning vector	$P_{\mathit{bla}_{\text{NDM-1}}}$, $\mathit{bla}_{\text{NDM-1}}$, P_{lac}, lacZ^{α}, P_{cat}, Cm^R, *ori*p15A	Gift from Prof. Matthew Avison
pYES2	Cloning vector	P_{GAL1}, $\mathit{CYC1}^{TT}$, *ori*pUC, Amp^R, P_{orif1}, $\mathit{ori}2\mu$, *URA*3	Lab stock
pYES–mt-11S	Expression vector	pYES carries *11S* gene with yeast $F_1\alpha$ signal sequence	This study

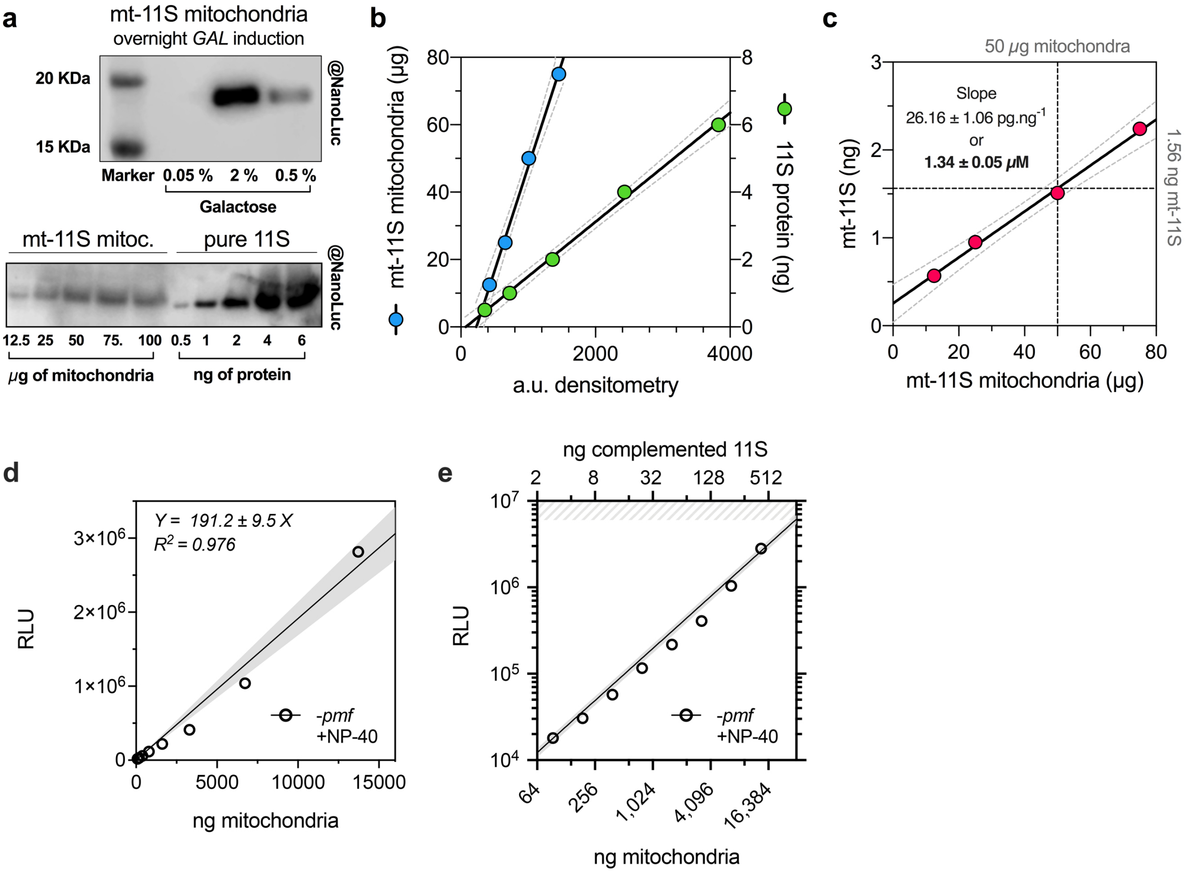

Figure S5: Detection of mt-11S and quantification in isolated mitochondria. mt-11S mitochondria were isolated by standard differential centrifugation and their protein content was then resolved by SDS-PAGE. 11S was detected using a primary antibody against NanoLuc (gift from Promega, US). **a** top – increasing amounts of mt-11S can be achieved by controling the amount of galactose during overnight induction; bottom – titration of mt-11S isolated mitochondrial fractions (induced at 1 % galactose) and $_{6H}$11S protein for quantification. **b** standard curves for 11S chemiluminescence signal as a function of mitochondrial or protein amount. **c** extrapolation of 11S in mt-11S mitochondria – there is 1.34 µM 11S per mitochondria. **d**, **e** relationship between signal of complemented 11S and amount of mitochondria. **e** is a $\log_{10}$-$\log_2$ plot of the linear plot on 'd'. For the experiments in 'd, e' a serial dilution (1:1) of mt-11S mitochondria was prepared in normal reaction buffer in the absence of import (DECA) and GST-dark but in the presence of the detergent NP-40, which releases the content of the mitochondria. The concentration of CytB2$_{\Delta 43\text{-}65}$-pep86 was was chosen to saturate the 11S, 1 µM. The reaction was allowed to equilibrate for 10 min before the addition of furimazine and measurement on the plate reader. Experimental data are shown as mean ± 95% confidence intervals. Dashed area on 'e' represent the limit of detection of the plate reader used (6 million RLU). n=1.

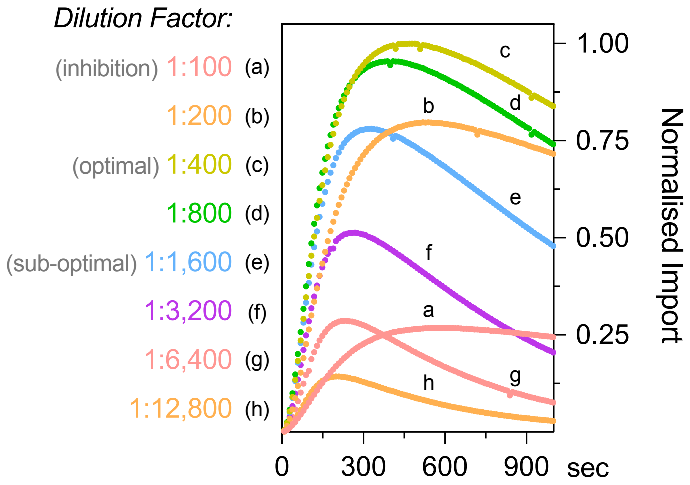

Figure S6: Inhibitory effect of Furimazine on mitochondrial import. Import of CytB2$_{\Delta 43\text{-}65}$-DHFR-pep86 into fully energised 11S-mitochondria in the presence of different concentrations of Furimazine (Fz). The manufacturer's recommended concentration is 1:100 (NanoGlo kit, Promega). Optimal concentration (1:400) was used in this study. n=1.

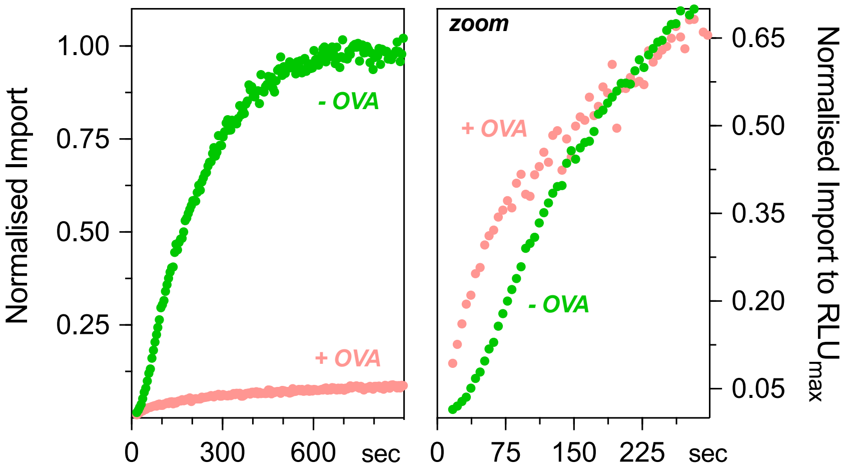

Figure S7: Distinction between import and background traces. Import of CytB2$_{\Delta 43\text{-}65}$-pep86 into NADH-only energised 11S-mitochondria in the absence (green) or presence (coral) of oligomycin, valinomycin and antimycin AA (OVA) cocktail – full *pmf* dissipation. In the absence of OVA (green) the curve is sigmoidal due to the presence of a lag period during the intial phase of import. Contrarily, the background trace (coral) experiences no lag and resembles a single exponential curve. On the left panel, import was normalised to the maximum RLU of each trace in order to make differences in lag easier to observe. n=1.

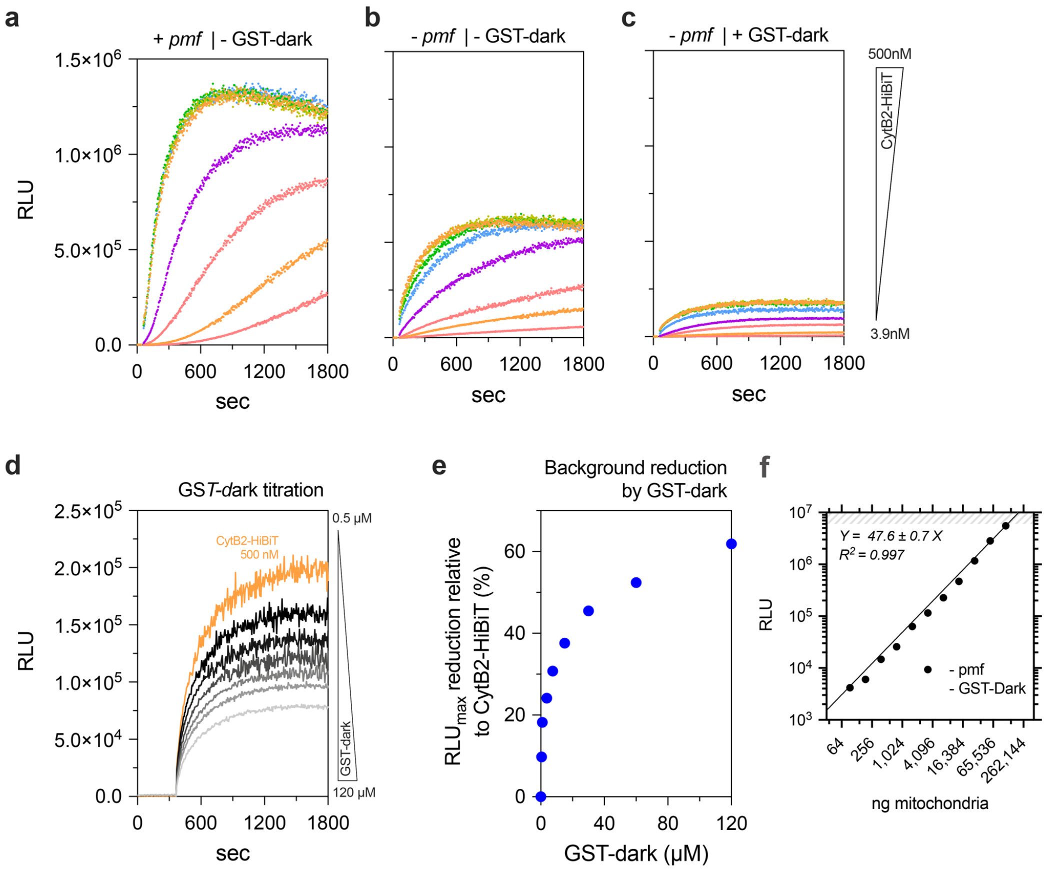

Figure S8: Panel **a** and **b** shows the import of CytB2$_{\Delta 43\text{-}65}$-pep86 into 11S mitochondria in the presence and absence of *pmf*, respectively. Observationaly, the background signal corresponds to about 1/3 of the RLU_{max}, independently of the concentration of preprotein-pep86 used. Because the signal is observed in the absence of *pmf*, the driving force for protein import, it reflects extra-mitochondrial events. This is supported by the observation that incubation of *GST-dark* in the absence of *pmf* (panel **c**) further reduces the background to about 20 % of the RLU_{max} in the presence of *pmf*. Panel **d** show a serial dilution of GST-dark in the presence of a constant concentration of CytB2$_{\Delta 43\text{-}65}$-pep86 (500 nM) in absence of *pmf*. RLU_{max} values from '**d**' are plotted on the graph in **e**. The data show that the GST-dark can decrease the background signal by ~ 50 % if used at a concentration 10x higher than the preprotein-pep86 used. **f** – relationship between the background signal in the absence of *pmf* and GST-dark as a function of the amount of mitochondria in the reaction. For the experiments in 'f' a serial dilution (1:1) of mt-11S mitochondria was prepared in normal reaction buffer in the absence of *pmf* and GST-dark. The concentration of CytB2$_{\Delta 43\text{-}65}$-pep86 was was chosen to saturate the 11S, 1 µM. The reaction was allowed to equilibrate for 10 min before the addition of furimazine and measurement on the plate reader. Dashed area on 'f' represent the limit of detection of the plate reader used (6 million RLU). n=1.

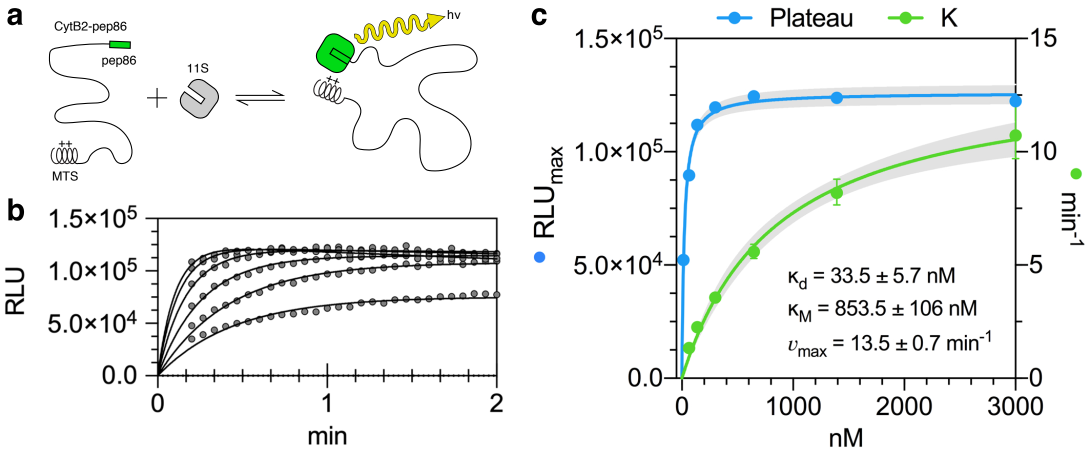

Figure S9: Pure CytB2$_{\Delta 43\text{-}65}$-pep86 and $_{6H}$11S complementation kinetics (**a**). To assess the binding affinity of preprotein-pep86 to 11S and its association constant, $_{6H}$11S was kept constant (30 pM) and and the preprotein-pep86 (CytB2$_{\Delta 43\text{-}65}$-pep86) varied from 3 μM to 46 nM. Experiments were setup on a 96 well plate by preparing a serial dilution of CytB2$_{\Delta 43\text{-}65}$-pep86 in NanoGlo buffer supplemented with furimazine following manufacturer's instructions (Promega). Then, complementation was started by injecting a fixed amount of $_{6H}$11S (25 μL) to achieve the desired final concentration in 125 μL. Luminescent signal was measured in a BioTek Synergy Neo2 every 4 sec simultaeously in 8 wells. Average of 2 independent runs are shown in panel **b**. Obtained data were fitted to a single exponential (black lines in **b**) and secondary data was plotted in the graph on panel **c**. Experimental data are shown as mean ± 95% confidence intervals. Error bars are not shown if smaller than the symbol. Fitted data are shown with the 95% confidence band in grey. n=2.

Table S5: Sequences of constructs used in this current study.

Name	Type	Sequence	Parameters
		signal sequence / mature protein / tags & protease cleave sites / pep86	
11S	DNA	1 ATGGTTTTCA CATTGGAGGA TTTTGTTGGG GATTGGGAAC AAACTGCAGC GTATAACTTG GATCAAGTCT TGGAACAAGG<br>81 TGGTGTTTCA TCCTTACTTC AGAATTTGGC TGTGTCTGTT ACACCTATTC AAAGGATAGT AAGATCAGGT GAAAATGCCC<br>161 TAAAGATCGA CATTCATGTC ATCATACCAT ATGAAGGTCT TAGTGCAGAT CAGATGGCAC AAATTGAGGA AGTCTTTAAA<br>241 GTCGTGTATC CAGTTGACGA TCATCACTTC AAAGTGATTT TACCCTATGG TACCTTAGTG ATAGATGGCG TTACACCTAA<br>321 CATGCTGAAC TACTTTGGCA GACCTTACGA AGGTATTGCT GTTTTCGATG GCAAGAAGAT AACTGTAACA GGAACTTTAT<br>401 GGAATGGCAA CAAAATCATT GACGAGAGGT TGATTACGCC AGATGGATCT ATGTTGTTTC GTGTAACCAT CAATAG	
	Protein	1 MVFTLEDFVG DWEQTAAYNL DQVLEQGGVS SLLQNLAVSV TPIQRIVRSG ENALKIDIHV IIPYEGLSAD QMAQIEEVFK<br>81 VVYPVDDHHF KVILPYGTLV IDGVTPNMLN YFGRPYEGIA VFDGKKITVT GTLWNGNKII DERLITPDGS MLFRVTINS	
$_{6H}$11S	DNA	1 ATGGGCCATC ATCATCATCA TCATGGCGTT TTCACATTGG AGGATTTTGT TGGGGATTGG GAACAAACTG CAGCGTATAA<br>81 CTTGGATCAA GTCTTGGAAC AAGGTGGTGT TTCATCCTTA CTTCAGAATT TGGCTGTGTC TGTTACACCT ATTCAAAGGA<br>161 TAGTAAGATC AGGTGAAAAT GCCCTAAAGA TCGACATTCA TGTCATCATA CCATATGAAG GTCTTAGTGC AGATCAGATG<br>241 GCACAAATTG AGGAAGTCTT TAAAGTCGTG TATCCAGTTG ACGATCATCA CTTCAAAGTG ATTTTACCCT ATGGTACCTT<br>321 AGTGATAGAT GGCGTTACAC CTAACATGCT GAACTACTTT GGCAGACCTT ACGAAGGTAT TGCTGTTTTC GATGGCAAGA<br>401 AGATAACTGT AACAGGAACT TTATGGAATG GCAACAAAAT CATTGACGAG AGGTTGATTA CGCCAGATGG ATCTATGTTG<br>481 TTTCGTGTAA CCATCAATAG CTAG	
	Protein	1 MGHHHHHGVF TLEDFVGDWE QTAAYNLDQV LEQGGVSSLL QNLAVSVTPI QRIVRSGENA LKIDIHVIIP YEGLSADQMA<br>81 QIEEVFKVVY PVDDHHFKVI LPYGTLVIDG VTPNMLNYFG RPYEGIAVFD GKKITVTGTL WNGNKIIDER LITPDGSMLF<br>161 RVTINS	
mt-11S	DNA	1 ATGGTGCTGG CGCGCACAGC CGCAATTCGC AGCTTAAGTC GGACGTTGAT TAACAGTACG AAAGCTGCTC GTCCGGCTGC<br>81 TGCAGCGCTC GCATCAACCC GTCGCTTGGC TAGCATGGTT TTCACATTGG AGGATTTTGT TGGGGATTGG GAACAAACTG<br>161 CAGCGTATAA CTTGGATCAA GTCTTGGAAC AAGGTGGTGT TTCATCCTTA CTTCAGAATT TGGCTGTGTC TGTTACACCT<br>241 ATTCAAAGGA TAGTAAGATC AGGTGAAAAT GCCCTAAAGA TCGACATTCA TGTCATCATA CCATATGAAG GTCTTAGTGC<br>321 AGATCAGATG GCACAAATTG AGGAAGTCTT TAAAGTCGTG TATCCAGTTG ACGATCATCA CTTCAAAGTG ATTTTACCCT<br>401 ATGGTACCTT AGTGATAGAT GGCGTTACAC CTAACATGCT GAACTACTTT GGCAGACCTT ACGAAGGTAT TGCTGTTTTC<br>481 GATGGCAAGA AGATAACTGT AACAGGAACT TTATGGAATG GCAACAAAAT CATTGACGAG AGGTTGATTA CGCCAGATGG<br>561 ATCTATGTTG TTTCGTGTAA CCATCAATAG	

continues on next page

Table S5 – Continued

signal sequence | mature protein | tags & protease cleave sites | pep86

	Protein	1 MVLARTAAIR SLSRTLINST KAARPAAAAL ASTRRLASMV FTLEDFVGDW EQTAAYNLDQ VLEQGGVSSL LQNLAVSVTP
		81 IQRIVRSGEN ALKIDIHVII PYEGLSADQM AQIEEVFKVV YPVDDHHFKV ILPYGTLVID GVTPNMLNYF GRPYEGIAVF
		161 DGKKITVTGT LWNGNKIIDE RLITPDGSML FRVTINS
NlpA-11S	DNA	1 ATGGGCAAAC TGACAACACA TCATCTACGG ACAGGGGCCG CATTATTGCT GGCCGGAATT CTGCTGGCAG GTTGCGACCA
		81 GAGTAGCAGC GGCGTTTTCA CATTGGAGGA TTTTGTTGGG GATTGGGAAC AAACTGCAGC GTATAACTTG GATCAAGTCT
		161 TGGAACAAGG TGGTGTTTCA TCCTTACTTC AGAATTTGGC TGTGTCTGTT ACACCTATTC AAAGGATAGT AAGATCAGGT
		241 GAAAATGCCC TAAAGATCGA CATTCATGTC ATCATACCAT ATGAAGGTCT TAGTGCAGAT CAGATGGCAC AAATTGAGGA
		321 AGTCTTTAAA GTCGTGTATC CAGTTGACGA TCATCACTTC AAAGTGATTT TACCCTATGG TACCTTAGTG ATAGATGGCG
		401 TTACACCTAA CATGCTGAAC TACTTTGGCA GACCTTACGA AGGTATTGCT GTTTTCGATG GCAAGAAGAT AACTGTAACA
		481 GGAACTTTAT GGAATGGCAA CAAAATCATT GACGAGAGGT TGATTACGCC AGATGGATCT ATGTTGTTTC GTGTAACCAT
		561 CAATAGCTAG
	Protein	1 MGKLTTHHLR TGAALLLAGI LLAGCDQSSS GVFTLEDFVG DWEQTAAYNL DQVLEQGGVS SLLQNLAVSV TPIQRIVRSG
		81 ENALKIDIHV IIPYEGLSAD QMAQIEEVFK VVYPVDDHHF KVILPYGTLV IDGVTPNMLN YFGRPYEGIA VFDGKKITVT
		161 GTLWNGNKII DERLITPDGS MLFRVTINS
pro-11S	DNA	1 ATGGGCAAAA AGACAGCTAT CGCGATTGCA GTGGCACTGG CTGGTTTCGC TACCGTAGCG CAGGCCGCTG GCGTTTTCAC
		81 ATTGGAGGTT TTCACATTGG AGGATTTTGT TGGGGATTGG GAACAAACTG CAGCGTATAA CTTGGATCAA GTCTTGGAAC
		161 AAGGTGGTGT TTCATCCTTA CTTCAGAATT TGGCTGTGTC TGTTACACCT ATTCAAAGGA TAGTAAGATC AGGTGAAAAT
		241 GCCCTAAAGA TCGACATTCA TGTCATCATA CCATATGAAG GTCTTAGTGC AGATCAGATG GCACAAATTG AGGAAGTCTT
		321 TAAAGTCGTG TATCCAGTTG ACGATCATCA CTTCAAAGTG ATTTTACCCT ATGGTACCTT AGTGATAGAT GGCGTTACAC
		401 CTAACATGCT GAACTACTTT GGCAGACCTT ACGAAGGTAT TGCTGTTTTC GATGGCAAGA AGATAACTGT AACAGGAACT
		481 TTATGGAATG GCAACAAAAT CATTGACGAG AGGTTGATTA CGCCAGATGG ATCTATGTTG TTTCGTGTAA CCATCAATAG
		561 CTAG
	Protein	1 MGKKTAIAIA VALAGFATVA QAAGVFTLED FVGDWEQTAA YNLDQVLEQG GVSSLLQNLA VSVTPIQRIV RSGENALKID
		81 IHVIIPYEGL SADQMAQIEE VFKVVYPVDD HHFKVILPYG TLVIDGVTPN MLNYFGRPYE GIAVFDGKKI TVTGTLWNGN
		161 KIIDERLITP DGSMLFRVTI NS

continues on next page

Table S5 – Continued

signal sequence | mature protein | tags & protease cleave sites | pep86

proSpy	DNA	1	ATGCGTAAAT TAACTGCACT GTTTGTTGCC TCTACCCTGG CTCTTGGCGC GGCTAACCTG GCCCATGCCG CAGACACCAC
		81	TACCGCAGCA CCGGCTGACG CGAAGCCGAT GATGCACCAC AAAGGCAAGT TCGGTCCGCA TCAGGACATG ATGTTCAAAG
		161	ACCTGAACCT GACCGACGCG CAGAAACAGC AGATCCGCGA AATCATGAAA GGCCAGCGTG ACCAGATGAA ACGTCCGCCG
		241	CTGGAAGAAC GCCGCGCAAT GCATGACATC ATTGCCAGCG ATACCTTCGA TAAAGTAAAA GCTGAAGCGC AGATCGCAAA
		321	AATGGAAGAA CAGCGCAAAG CTAACATGCT GGCGCACATG GAAACCCAGA ACAAAATTTA CAACATCCTG ACGCCGGAAC
		401	AGAAAAAGCA ATTTAATGCT AATTTTGAGA AGCGTCTGAC AGAACGTCCA GCGGCAAAAG GTAAAATGCC TGCAACTGCT
		481	GAAATTCCGA ATCCGCTGCT GGGTCTGGAA AACCTGTATT TTCAGGGCCA TCATCATCAT CATCATTAA
	Protein	1	MRKLTALFVA STLALGAANL AHAADTTTAA PADAKPMMHH KGKFGPHQDM MFKDLNLTDA QKQQIREIMK GQRDQMKRPP
		81	LEERRAMHDI IASDTFDKVK AEAQIAKMEE QRKANMLAHM ETQNKIYNIL TPEQKKQFNA NFEKRLTERP AAKGKMPATA
		161	EIPNPLLGLE NLYFQGHHHH HH
proSpy-pep86	DNA	1	ATGCGTAAAT TAACTGCACT GTTTGTTGCC TCTACCCTGG CTCTTGGCGC GGCTAACCTG GCCCATGCCG CAGACACCAC
		81	TACCGCAGCA CCGGCTGACG CGAAGCCGAT GATGCACCAC AAAGGCAAGT TCGGTCCGCA TCAGGACATG ATGTTCAAAG
		161	ACCTGAACCT GACCGACGCG CAGAAACAGC AGATCCGCGA AATCATGAAA GGCCAGCGTG ACCAGATGAA ACGTCCGCCG
		241	CTGGAAGAAC GCCGCGCAAT GCATGACATC ATTGCCAGCG ATACCTTCGA TAAAGTAAAA GCTGAAGCGC AGATCGCAAA
		321	AATGGAAGAA CAGCGCAAAG CTAACATGCT GGCGCACATG GAAACCCAGA ACAAAATTTA CAACATCCTG ACGCCGGAAC
		401	AGAAAAAGCA ATTTAATGCT AATTTTGAGA AGCGTCTGAC AGAACGTCCA GCGGCAAAAG GTAAAATGCC TGCAACTGCT
		481	GAAATTCCGA ATCCGCTGCT GGGTCTGGGC TCCGGCGTGA GCGGCTGGCG CCTGTTTAAA AAAATTAGCG AAAACCTGTA
		561	TTTTCAGGGC CATCATCATC ATCATCATTA A
	Protein	1	MRKLTALFVA STLALGAANL AHAADTTTAA PADAKPMMHH KGKFGPHQDM MFKDLNLTDA QKQQIREIMK GQRDQMKRPP
		81	LEERRAMHDI IASDTFDKVK AEAQIAKMEE QRKANMLAHM ETQNKIYNIL TPEQKKQFNA NFEKRLTERP AAKGKMPATA
		161	EIPNPLLGLG SGVSGWRLFK KISENLYFQG HHHHHH
mSpy	DNA	1	ATGGCAGACA CCACTACCGC AGCACCGGCT GACGCGAAGC CGATGATGCA CCACAAAGGC AAGTTCGGTC CGCATCAGGA
		81	CATGATGTTC AAAGACCTGA ACCTGACCGA CGCGCAGAAA CAGCAGATCC GCGAAATCAT GAAAGGCCAG CGTGACCAGA
		161	TGAAACGTCC GCCGCTGGAA GAACGCCGCG CAATGCATGA CATCATTGCC AGCGATACCT TCGATAAAGT AAAAGCTGAA
		241	GCGCAGATCG CAAAAATGGA AGAACAGCGC AAAGCTAACA TGCTGGCGCA CATGGAAACC CAGAACAAAA TTTACAACAT
		321	CCTGACGCCG GAACAGAAAA AGCAATTTAA TGCTAATTTT GAGAAGCGTC TGACAGAACG TCCAGCGGCA AAAGGTAAAA
		401	TGCCTGCAAC TGCTGAAATT CCGAATCCGC TGCTGGGTCT GGAAAACCTG TATTTTCAGG GCCATCATCA TCATCATCAT
		481	TAA

continues on next page

Table S5 – Continued

signal sequence | mature protein | tags & protease cleave sites | pep86

	Protein	1	MADTTTAAPA DAKPMMHHKG KFGPHQDMMF KDLNLTDAQK QQIREIMKGQ RDQMKRPPLE ERRAMHDIIA SDTFDKVKAE
		81	AQIAKMEEQR KANMLAHMET QNKIYNILTP EQKKQFNANF EKRLTERPAA KGKMPATAEI PNPLLGLENL YFQGHHHHHH
mSpy-pep86	DNA	1	ATGGCAGACA CCACTACCGC AGCACCGGCT GACGCGAAGC CGATGATGCA CCACAAAGGC AAGTTCGGTC CGCATCAGGA
		81	CATGATGTTC AAAGACCTGA ACCTGACCGA CGCGCAGAAA CAGCAGATCC GCGAAATCAT GAAAGGCCAG CGTGACCAGA
		161	TGAAACGTCC GCCGCTGGAA GAACGCCGCG CAATGCATGA CATCATTGCC AGCGATACCT TCGATAAAGT AAAAGCTGAA
		241	GCGCAGATCG CAAAAATGGA AGAACAGCGC AAAGCTAACA TGCTGGCGCA CATGGAAACC CAGAACAAAA TTTACAACAT
		321	CCTGACGCCG GAACAGAAAA AGCAATTTAA TGCTAATTTT GAGAAGCGTC TGACAGAACG TCCAGCGGCA AAAGGTAAAA
		401	TGCCTGCAAC TGCTGAAATT CCGAATCCGC TGCTGGGTCT GGGTTCTGGG GTAAGTGGTT GGCGCTTGTT CAAGAAAATC
		481	TCCGAAAACC TGTATTTTCA GGGCCATCAT CATCATCATC ATTAA
	Protein	1	MADTTTAAPA DAKPMMHHKG KFGPHQDMMF KDLNLTDAQK QQIREIMKGQ RDQMKRPPLE ERRAMHDIIA SDTFDKVKAE
		81	AQIAKMEEQR KANMLAHMET QNKIYNILTP EQKKQFNANF EKRLTERPAA KGKMPATAEI PNPLLGLGSG VSGWRLFKKI
		161	SENLYFQGHH HHHH
proOmpA	DNA	1	ATGAAAAAGA CAGCTATCGC GATTGCAGTG GCACTGGCTG GTTTCGCTAC CGTAGCGCAG GCCGCTCCGA AAGATAACAC
		81	CTGGTACACT GGTGCTAAAC TGGGCTGGTC CCAGTACCAT GATACTGGTT TCATCAACAA CAATGGCCCG ACCCATGAAA
		161	ACCAACTGGG CGCTGGTGCT TTTGGTGGTT ACCAGGTTAA CCCGTATGTT GGCTTTGAAA TGGGTTACGA CTGGTTAGGT
		241	CGTATGCCGT ACAAAGGCAG CGTTGAAAAC GGTGCATACA AAGCTCAGGG CGTTCAACTG ACCGCTAAAC TGGGTTACCC
		321	AATCACTGAC GACCTGGACA TCTACACTCG TCTGGGTGGC ATGGTATGGC GTGCAGACAC TAAATCCAAC GTTTATGGTA
		401	AAAACCACGA CACCGGCGTT TCTCCGGTCT TCGCTGGCGG TGTTGAGTAC GCGATCACTC CTGAAATCGC TACCCGTCTG
		481	GAATACCAGT GGACGAACAA CATCGGTGAC GCACACACCA TCGGCACTCG TCCGGACAAC GGCATGCTGA GCCTGGGTGT
		561	TTCCTACCGT TTCGGTCAGG GCGAGGCAGC TCCAGTAGTT GCTCCGGCTC CAGCTCCGGC ACCGGAAGTA CAGACCAAGC
		641	ACTTCACTCT GAAGTCTGAC GTTCTGTTCA ACTTCAACAA AGCAACCCTG AAACCGGAAG GTCAGGCTGC TCTGGATCAG
		721	CTGTACAGCC AGCTGAGCAA CTTGGATCCG AAAGACGGTT CCGTAGTTGT TCTGGGTTAC ACCGACCGCA TCGGTTCTGA
		801	CGCTTACAAC CAGGGTCTGT CCGAGCGCCG TGCTCAGTCT GTTGTTGATT ACCTGATCTC CAAAGGTATC CCGGCAGACA
		881	AGATCTCCGC ACGTGGTATG GGCGAATCCA ACCCGGTTAC TGGCAACACC TCAGACAACG TGAAACAGCG TGCTGCACTG
		961	ATCGACTCAC TGGCTCCGGA TCGTCGCGTA GAGATCGAAG TTAAAGGTAT CAAAGACGTT GTAACTCAGC CGCAGGCTTG
		1041	GTCACATCCA CAATTCGAAA AATAA

continues on next page

Table S5 – Continued

signal sequence | mature protein | tags & protease cleave sites | pep86

		1	MKKTAIAIAV ALAGFATVAQ AAPKDNTWYT GAKLGWSQYH DTGFINNNGP THENQLGAGA FGGYQVNPYV GFEMGYDWLG
		81	RMPYKGSVEN GAYKAQGVQL TAKLGYPITD DLDIYTRLGG MVWRADTKSN VYGKNHDTGV SPVFAGGVEY AITPEIATRL
	Protein	161	EYQWTNNIGD AHTIGTRPDN GMLSLGVSYR FGQGEAAPVV APAPAPAPEV QTKHFTLKSD VLFNFNKATL KPEGQAALDQ
		241	LYSQLSNLDP KDGSVVVLGY TDRIGSDAYN QGLSERRAQS VVDYLISKGI PADKISARGM GESNPVTGNT SDNVKQRAAL
		321	IDSLAPDRRV EIEVKGIKDV VTQPQAWSHP QFEK
proOmpA-pep86	DNA	1	ATGGGCAAAA AGACAGCTAT CGCGATTGCA GTGGCACTGG CTGGTTTCGC TACCGTAGCG CAGGCCGCTC CGAAAGATAA
		81	CACCTGGTAC ACTGGTGCTA AACTGGGCTG GTCCCAGTAC CATGATACTG GTTTCATCAA CAACAATGGC CCGACCCATG
		161	AAAACCAACT GGGCGCTGGT GCTTTTGGTG GTTACCAGGT TAACCCGTAT GTTGGCTTTG AAATGGGTTA CGACTGGTTA
		241	GGTCGTATGC CGTACAAAGG CAGCGTTGAA AACGGTGCAT ACAAAGCTCA GGGCGTTCAA CTGACCGCTA AACTGGGTTA
		321	CCCAATCACT GACGACCTGG ACATCTACAC TCGTCTGGGT GGCATGGTAT GGCGTGCAGA CACTAAATCC AACGTTTATG
		401	GTAAAAACCA CGACACCGGC GTTTCTCCGG TCTTCGCTGG CGGTGTTGAG TACGCGATCA CTCCTGAAAT CGCTACCCGT
		481	CTGGAATACC AGTGGACGAA CAACATCGGT GACGCACACA CCATCGGCAC TCGTCCGGAC AACGGCATGC TGAGCCTGGG
		561	TGTTTCCTAC CGTTTCGGTC AGGGCGAGGC AGCTCAGTAG TTGCTCCGGC TCCAGCTCCG GCACCGGAAG TACAGACCAA
		641	GCACTTCACT CTGAAGTCTG ACGTTCTGTT CAACTTCAAC AAAGCAACCC TGAAACCGGA AGGTCAGGCT GCTCTGGATC
		721	AGCTGTACAG CCAGCTGAGC AACTTGGATC CGAAAGACGG TTCCGTAGTT GTTCTGGGTT ACACCGACCG CATCGGTTCT
		801	GACGCTTACA ACCAGGGTCT GTCCGAGCGC CGTGCTCAGT CTGTTGTTGA TTACCTGATC TCCAAAGGTA TCCCGGCAGA
		881	CAAGATCTCC GCACGTGGTA TGGGCGAATC CAACCCGGTT ACTGGCAACA CCTCAGACAA CGTGAAACAG CGTGCTGCAC
		961	TGATCGACTC ACTGGCTCCG GATCGTCGCG TAGAGATCGA AGTTAAAGGT ATCAAAGACG TTGTAACTCA GCCGCAGGCT
		1041	TGGTCACATC CACAATTCGA AAAAGGCTCC GGCGTGAGCG GCTGGCGCCT GTTTAAAAAA ATTAGCTAA
		1	MKKTAIAIAV ALAGFATVAQ AAPKDNTWYT GAKLGWSQYH DTGFINNNGP THENQLGAGA FGGYQVNPYV GFEMGYDWLG
		81	RMPYKGSVEN GAYKAQGVQL TAKLGYPITD DLDIYTRLGG MVWRADTKSN VYGKNHDTGV SPVFAGGVEY AITPEIATRL
	Protein	161	EYQWTNNIGD AHTIGTRPDN GMLSLGVSYR FGQGEAAPVV APAPAPAPEV QTKHFTLKSD VLFNFNKATL KPEGQAALDQ
		241	LYSQLSNLDP KDGSVVVLGY TDRIGSDAYN QGLSERRAQS VVDYLISKGI PADKISARGM GESNPVTGNT SDNVKQRAAL
		321	IDSLAPDRRV EIEVKGIKDV VTQPQAWSHP QFEKGSGVSG WRLFKKIS

continues on next page

Table S5 – Continued

signal sequence · mature protein · tags & protease cleave sites · pep86

CytB2$_{\Delta 43\text{-}65}$	DNA	1 ATGGTGAAAT ACAAACCGTT GCTGAAAATC TCAAAGAACA GTGAAGCGGC CATTTTACGG GCGTCTAAAA CCCGCTTAAA
		81 CACCATTCGC GCTTATGGCA GCACTGTTCC GAAAAGCAAA TCGTTTTCGT CAGTGGCATA CCTGAATTGG CACAATGGTC
		161 AAATTGACAA TGAACCAAAA CTGGACATGA ACAAACAGAA AATTTCCCCT GCAGAAGTAG CGAAACACAA CAAACCGGAT
		241 GACTCTTGGG TAGTAATCAA TGGATATGTC TATGATCTTA CCCGTTTTCT GCCGAACCAT CCAGGAGGGC AGGACGTCAT
		321 CAAGTTCAAC GCGGGTAAAG ATGTGACAGC CATCTTTGAA CCGCTCCATG CACCCAATGT GATCGATAAA TATATTGCAC
		401 CGGAGGGTAC CTTGGTGAGC GAAAACCTGT ACTTTCAAGG CGGCTCCGGT GAAAACTTGT ATTTCCAAGG TGGTGGGTTA
		481 GTATCAGAGC AGAAACTGAT TTCCGAAGAG GATCTGCTGC AGCATCATCA CCATCATCAC TAG
	Protein	1 MVKYKPLLKI SKNSEAAILR ASKTRLNTIR AYGSTVPKSK SFSSVAYLNW HNGQIDNEPK LDMNKQKISP AEVAKHNKPD
		81 DSWVVINGYV YDLTRFLPNH PGGQDVIKFN AGKDVTAIFE PLHAPNVIDK YIAPEGTLVS ENLYFQGGSG ENLYFQGGGL
		161 VSEQKLISEE DLLQHHHHHH
CytB2$_{\Delta 43\text{-}65}$-pep114	DNA	1 MVKYKPLLKI SKNSEAAILR ASKTRLNTIR AYGSTVPKSK SFSSVAYLNW HNGQIDNEPK LDMNKQKISP AEVAKHNKPD
		81 DSWVVINGYV YDLTRFLPNH PGGQDVIKFN AGKDVTAIFE PLHAPNVIDK YIAPEGTLVS ENLYFQGGSG ENLYFQGGGL
		161 VSEQKLISEE DLLQHHHHHH SGGGGSVTGY RLFEEIL
	Protein	1 MVKYKPLLKI SKNSEAAILR ASKTRLNTIR AYGSTVPKSK SFSSVAYLNW HNGQIDNEPK LDMNKQKISP AEVAKHNKPD
		81 DSWVVINGYV YDLTRFLPNH PGGQDVIKFN AGKDVTAIFE PLHAPNVIDK YIAPEGTLVS ENLYFQGGSG ENLYFQGGGL
		161 VSEQKLISEE DLLQHHHHHH SGGGGSVSGW RLFKKIS
CytB2$_{\Delta 43\text{-}65}$-pep86	DNA	1 ATGGTGAAAT ACAAACCCTT GCTTAAGATT TCCAAGAATT GCGAAGCAGC GATTTTACGC GCGAGCAAAA CCCGGTTAAA
		81 TACCATTCGT GCGTATGGCT CGACCGTGCC AAAATCCAAA TCGTTCTCTA GTGTTGCGTA TCTGAATTGG CATAACGGCC
		161 AGATTGACAA TGAACCGAAA CTCGACATGA ACAAACAGAA GATCTCACCC GCAGAAGTAG CCAAACACAA CAAACCGGAT
		241 GATTGCTGGG TGGTCATTAA CGGCTATGTC TATGACTTGA CTCGCTTTCT CCCGAATCAT CCTGGAGGCC AGGATGTGAT
		321 CAAATTCAAT GCTGGAAAAG ATGTGACAGC CATCTTTGAG CCGCTGCATG CGCCGAACGT CATTGACAAA TACATTGCTC
		401 CGGAAAAGAA ACTGGGTCCG CTGCAAGGCT CTATGCCTCC AGAACTCGTT TGTCTCCGTA TGCACCAGGG GAAACGAAAG
		481 AGGATATTGC GCGTAAAGAG CAGCTGAAAA GCCTGTTGCC GCCGTTAGAC AACATCATCA ACCTTTATGA TTTCGAATAC
		561 CTGGCTAGCC AAACGCTGAC CAAACAGGCC GAACAGAAGC TGATCAGCGA AGAGGATCTG CATCATCACC ATCATCACGG
		641 TGGAGGCAGT GTGTCTGGTT GGCGTCTGTT CAAGAAAATT TCGTAG
	Protein	1 MVKYKPLLKI SKNSEAAILR ASKTRLNTIR AYGSTVPKSK SFSSVAYLNW HNGQIDNEPK LDMNKQKISP AEVAKHNKPD
		81 DCWVVINGYV YDLTRFLPNH PGGQDVIKFN AGKDVTAIFE PLHAPNVIDK YIAPEKKLGP LQGSMPPELV CPPYAPGETK
		161 EDIARKEQLK SLLPPLDNII NLYDFEYLAS QTLTKQAEQK LISEEDLHHH HHHGGGSVSG WRLFKKIS

continues on next page

Table S5 – Continued

signal sequence | mature protein | tags & protease cleave sites | pep86

Construct	Type	Position	Sequence
CytB2$_{\Delta 43\text{-}65\ \Delta 2\text{-}20}$-pep86	Protein	1	ATGGCGAGCA AAACCCGGTT AAATACCATT CGTGCGTATG GCTCGACCGT GCCAAAATCC AAATCGTTCT CTAGTGTTGC
		81	GTATCTGAAT TGGCATAACG GCCAGATTGA CAATGAACCG AAACTCGACA TGAACAAACA GAAGATCTCA CCCGCAGAAG
		161	TAGCCAAACA CAACAAACCG GATGATTGCT GGGTGGTCAT TAACGGCTAT GTCTATGACT TGACTCGCTT TCTCCCGAAT
		241	CATCCTGGAG GCCAGGATGT GATCAAATTC AATGCTGGAA AAGATGTGAC AGCCATCTTT GAGCCGCTGC ATGCGCCGAA
		321	CGTCATTGAC AAATACATTG CTCCGGAAAA GAAACTGGGT CCGCTGCAAG GCTCTATGCC TCCAGAACTC GTTTGTCCTC
		401	CGTATGCACC AGGGGAAACG AAAGAGGATA TTGCGCGTAA AGAGCAGCTG AAAAGCCTGT TGCCGCCGTT AGACAACATC
		481	ATCAACCTTT ATGATTTCGA ATACCTGGCT AGCCAAACGC TGACCAAACA GGCCGAACAG AAGCTGATCA GCGAAGAGGA
		561	TCTGCATCAT CACCATCATC ACGGTGGAGG CAGTGTGTCT GGTTGGCGTC TGTTCAAGAA AATTTCGTAG
CytB2$_{\Delta 43\text{-}65\ \Delta 2\text{-}20}$-pep86	Protein	1	M--------- ---------R ASKTRLNTIR AYGSTVPKSK SFSSVAYLNW HNGQIDNEPK LDMNKQKISP AEVAKHNKPD
		81	DCWVVINGYV YDLTRFLPNH PGGQDVIKFN AGKDVTAIFE PLHAPNVIDK YIAPEKKLGP LQGSMPPELV CPPYAPGETK
		161	EDIARKEQLK SLLPPLDNII NLYDFEYLAS QTLTKQAEQK LISEEDLHHH HHHGGGSVSG WRLFKKIS
CytB2$_{\Delta 2\text{-}65}$-pep86	DNA	1	ATGTCTAGTG TTGCGTATCT GAATTGGCAT AACGGCCAGA TTGACAATGA ACCGAAACTC GACATGAACA AACAGAAGAT
		81	CTCACCCGCA GAAGTAGCCA AACACAACAA ACCGGATGAT TGCTGGGTGG TCATTAACGG CTATGTCTAT GACTTGACTC
		161	GCTTTCTCCC GAATCATCCT GGAGGCCAGG ATGTGATCAA ATTCAATGCT GGAAAAGATG TGACAGCCAT CTTTGAGCCG
		241	CTGCATGCGC CGAACGTCAT TGACAAATAC ATTGCTCCGG AAAAGAAACT GGGTCCGCTG CAAGGCTCTA TGCCTCCAGA
		321	ACTCGTTTGT CCTCCGTATG CACCAGGGGA AACGAAAGAG GATATTGCGC GTAAAGAGCA GCTGAAAAGC CTGTTGCCGC
		401	CGTTAGACAA CATCATCAAC CTTTATGATT TCGAATACCT GGCTAGCCAA ACGCTGACCA AACAGGCCGA ACAGAAGCTG
		481	ATCAGCGAAG AGGATCTGCA TCATCACCAT CATCACGGTG GAGGCAGTGT GTCTGGTTGG CGTCTGTTCA AGAAAATTTC
		561	GTAG
	Protein	1	M--------- ---------- ---------- ---------- --SSVAYLNW HNGQIDNEPK LDMNKQKISP AEVAKHNKPD
		81	DCWVVINGYV YDLTRFLPNH PGGQDVIKFN AGKDVTAIFE PLHAPNVIDK YIAPEKKLGP LQGSMPPELV CPPYAPGETK
		161	EDIARKEQLK SLLPPLDNII NLYDFEYLAS QTLTKQAEQK LISEEDLHHH HHHGGGSVSG WRLFKKIS
CytB2$_{\Delta 2\text{-}80}$-pep86	DNA	1	ATGGAACCGA AACTCGACAT GAACAAACAG AAGATCTCAC CCGCAGAAGT AGCCAAACAC AACAAACCGG ATGATTGCTG
		81	GGTGGTCATT AACGGCTATG TCTATGACTT GACTCGCTTT CTCCCGAATC ATCCTGGAGG CCAGGATGTG ATCAAATTCA
		161	ATGCTGGAAA AGATGTGACA GCCATCTTTG AGCCGCTGCA TGCGCCGAAC GTCATTGACA AATACATTGC TCCGGAAAAG
		241	AAACTGGGTC CGCTGCAAGG CTCTATGCCT CCAGAACTCG TTTGTCCTCC GTATGCACCA GGGGAAACGA AAGAGGATAT
		321	TGCGCGTAAA GAGCAGCTGA AAAGCCTGTT GCCGCCGTTA GACAACATCA TCAACCTTTA TGATTTCGAA TACCTGGCTA
		401	GCCAAACGCT GACCAAACAG GCCGAACAGA AGCTGATCAG CGAAGAGGAT CTGCATCATC ACCATCATCA CGGTGGAGGC
		481	AGTGTGTCTG GTTGGCGTCT GTTCAAGAAA ATTTCGTAG

continues on next page

Table S5 – Continued

signal sequence | mature protein | tags & protease cleave sites | pep86

	1	M--------- ---------- ---------- ---------- ---------- -------EPK LDMNKQKISP AEVAKHNKPD
Protein	81	DCWVVINGYV YDLTRFLPNH PGGQDVIKFN AGKDVTAIFE PLHAPNVIDK YIAPEKKLGP LQGSMPPELV CPPYAPGETK
	161	EDIARKEQLK SLLPPLDNII NLYDFEYLAS QTLTKQAEQK LISEEDLHHH HHHGGGSVSG WRLFKKIS

[1] C. T. Chung, S. L. Niemela, R. H. Miller, One-step preparation of competent escherichia coli: transformation and storage of bacterial cells in the same solution, Proceedings of the National Academy of Sciences 86 (1989) 2172–2175. doi:10.1073/pnas.86.7.2172.

[2] R. D. Gietz, R. H. Schiestl, High-efficiency yeast transformation using the liac/ss carrier dna/peg method, Nature Protocols 2 (2007) 31–34.

[3] A. S. Dixon, M. K. Schwinn, M. P. Hall, K. Zimmerman, P. Otto, T. H. Lubben, B. L. Butler, B. F. Binkowski, T. Machleidt, T. A. Kirkland, M. G. Wood, C. T. Eggers, L. P. Encell, K. V. Wood, Nanoluc complementation reporter optimized for accurate measurement of protein interactions in cells, ACS Chemical Biology 11 (2016) 400–408. doi:10.1021/acschembio.5b00753.

[4] B. R. Harvey, G. Georgiou, A. Hayhurst, K. J. Jeong, B. L. Iverson, G. K. Rogers, Anchored periplasmic expression, a versatile technology for the isolation of high-affinity antibodies from escherichia coli-expressed libraries, Proceedings of the National Academy of Sciences 101 (2004) 9193–9198. doi:10.1073/pnas.0400187101.

[5] D. A. S, E. Lance, H. Mary, W. Keith, W. Monika, S. Marie, B. B. F, Z. Hicham, N. Nidhi, M. Subhanjan, G. Said, M. Poncho, K. Thomas, U. James, P. D. K, R. Matthew, D. Melanie, M. Thomas, Activation of bioluminescence by structural complementation, ???? URL: https://lens.org/104-315-079-589-898.

[6] R. A. Corey, E. Pyle, W. J. Allen, D. W. Watkins, M. Casiraghi, B. Miroux, I. Arechaga, A. Politis, I. Collinson, Specific cardiolipin–secy interactions are required for proton-motive force stimulation of protein secretion, Proceedings of the National Academy of Sciences 115 (2018) 7967–7972. doi:10.1073/pnas.1721536115.

[7] V. A. M. Gold, R. Ieva, A. Walter, N. Pfanner, M. van der Laan, W. Kühlbrandt, Visualizing active membrane protein complexes by electron cryotomography, Nature Communications 5 (2014) 4129.

[8] D. B. Oliver, J. Beckwith, E. coli mutant pleiotropically defective in the export of secreted proteins, Cell 25 (1981) 765–772. doi:10.1016/0092-8674(81)90184-7.

[9] B. Ballhausen, K. Altendorf, G. Deckers-Hebestreit, Constant c10 ring stoichiometry in the escherichia coli atp synthase analyzed by cross-linking, Journal of Bacteriology 191 (2009) 2400–2404. doi:10.1128/JB.01390-08.

[10] S. Backes, S. Hess, F. Boos, M. W. Woellhaf, S. Gödel, M. Jung, T. Mühlhaus, J. M. Herrmann, Tom70 enhances mitochondrial preprotein import efficiency by binding to internal targeting sequences, The Journal of Cell Biology 217 (2018) 1369–1382. doi:10.1083/jcb.201708044.

[11] T. S. Wehrman, C. L. Casipit, N. M. Gewertz, H. M. Blau, Enzymatic detection of protein translocation, Nature methods 2 (2005) 521.

[12] C. J. Smoyer, S. S. Katta, J. M. Gardner, L. Stoltz, S. McCroskey, W. D. Bradford, M. McClain, S. E. Smith, B. D. Slaughter, J. R. Unruh, et al., Analysis of membrane proteins localizing to the inner nuclear envelope in living cells, J Cell Biol (2016) jcb–201607043.

[13] A. Patel, J. Murray, S. McElwee-Whitmer, C. Bai, P. Kunapuli, E. N. Johnson, A combination of ultrahigh throughput pathhunter and cytokine secretion assays to identify glucocn.zc.nc.vzzzticoid receptor agonists, Analytical biochemistry 385 (2009) 286–292.

[14] G. Calmettes, J. N. Weiss, A quantitative method to track protein translocation between intracellular compartments in real-time in live cells using weighted local variance image analysis, PloS one 8 (2013) e81988.

[15] M. Krayl, B. Guiard, K. Paal, W. Voos, Fluorescence-mediated analysis of mitochondrial preprotein import in vitro, Analytical biochemistry 355 (2006) 81–89.

[16] F.-C. Liang, U. K. Bageshwar, S. M. Musser, Bacterial sec protein transport is rate-limited by precursor length: a single turnover study, Molecular biology of the cell 20 (2009) 4256–4266.

[17] T. Ozawa, Y. Sako, M. Sato, T. Kitamura, Y. Umezawa, A genetic approach to identifying mitochondrial proteins, Nature biotechnology 21 (2003) 287.

[18] T. Ozawa, Y. Natori, Y. Sako, H. Kuroiwa, T. Kuroiwa, Y. Umezawa, A minimal peptide sequence that targets fluorescent and functional proteins into the mitochondrial intermembrane space, ACS chemical biology 2 (2007) 176–186.

[19] H.-W. Rhee, P. Zou, N. D. Udeshi, J. D. Martell, V. K. Mootha, S. A. Carr, A. Y. Ting, Proteomic mapping of mitochondria in living cells via spatially restricted enzymatic tagging, Science 339 (2013) 1328–1331.

[20] K. Hoogewijs, A. M. James, R. A. Smith, M. J. Gait, M. P. Murphy, R. N. Lightowlers, Assessing the delivery of molecules to the mitochondrial matrix using click chemistry, Chembiochem 17 (2016) 1312–1316.
